# The identification of the new species *Nitratireductor thuwali* sp. nov. reveals the untapped diversity of hydrocarbon-degrading culturable bacteria from the arid mangrove sediments of the Red Sea

**DOI:** 10.3389/fmicb.2023.1155381

**Published:** 2023-05-02

**Authors:** Ramona Marasco, Grégoire Michoud, Fatmah O. Sefrji, Marco Fusi, Chakkiath P. Antony, Kholoud A. Seferji, Alan Barozzi, Giuseppe Merlino, Daniele Daffonchio

**Affiliations:** Red Sea Research Center, Biological and Environmental Sciences and Engineering Division (BESE), King Abdullah University of Science and Technology (KAUST), Thuwal, Saudi Arabia

**Keywords:** heterotrophic bacteria, cultivation, mangroves, extreme adaptation, rare biosphere, petroleum hydrocarbon-degraders

## Abstract

**Introduction:**

The geological isolation, lack of freshwater inputs and specific internal water circulations make the Red Sea one of the most extreme—and unique—oceans on the planet. Its high temperature, salinity and oligotrophy, along with the consistent input of hydrocarbons due to its geology (e.g., deep-sea vents) and high oil tankers traffic, create the conditions that can drive and influence the assembly of unique marine (micro)biomes that evolved to cope with these multiple stressors. We hypothesize that mangrove sediments, as a model-specific marine environment of the Red Sea, act as microbial hotspots/reservoirs of such diversity not yet explored and described.

**Methods:**

To test our hypothesis, we combined oligotrophic media to mimic the Red Sea conditions and hydrocarbons as C-source (i.e., crude oil) with long incubation time to allow the cultivation of slow-growing environmentally (rare or uncommon) relevant bacteria.

**Results and discussion:**

This approach reveals the vast diversity of taxonomically novel microbial hydrocarbon degraders within a collection of a few hundred isolates. Among these isolates, we characterized a novel species, *Nitratireductor thuwali* sp. nov., namely, Nit1536^T^. It is an aerobic, heterotrophic, Gram-stain-negative bacterium with optimum growth at 37°C, 8 pH and 4% NaCl, whose genome and physiological analysis confirmed the adaptation to extreme and oligotrophic conditions of the Red Sea mangrove sediments. For instance, Nit1536^T^ metabolizes different carbon substrates, including straight-chain alkanes and organic acids, and synthesizes compatible solutes to survive in salty mangrove sediments. Our results showed that the Red Sea represent a source of yet unknown novel hydrocarbon degraders adapted to extreme marine conditions, and their discovery and characterization deserve further effort to unlock their biotechnological potential.

## Introduction

Mangroves are intertidal forests that play a crucial role in cycling energy and nutrients at the interface between land and sea (Sheaves, 2009; Donato et al., 2011), covering between 60 and 70% of the world’s tropical and subtropical coastlines (Thatoi et al., 2013). These productive ecosystems (Sheaves, 2009; Donato et al., 2011; Saderne et al., 2021) are characterized by periodic tidal flooding and fluctuating environmental factors, such as nutrients, oxygen, salinity and temperature (Fusi et al., 2022). The interaction of all these variables creates complex multidimensional habitats (Radhakrishnan et al., 2011) in which a huge number of microorganisms live, i.e., 91% of the total microbial biomass (Pramanik et al., 2019). Located on the coasts, mangroves have also to withstand the continuous pressure of the growing anthropogenic activities (Allard et al., 2020; Martin et al., 2020; Adyasari et al., 2021; Luo et al., 2021), including urbanization and aquaculture farming (Maiti and Chowdhury, 2013; Fusi et al., 2016; Richards and Friess, 2016). These activities impact mangroves with sewage, antibiotics, pesticides, microplastic and other contaminants, such as hydrocarbons released by intense ship traffic and numerous deep-sea vents (Andreote et al., 2012; Maiti and Chowdhury, 2013; Richards and Friess, 2016; El-Amin Bashir et al., 2017; Alzahrani et al., 2018; Martin et al., 2019b,^2020^). Stressful conditions can also be induced by other abiotic factors, such as lack of freshwater input, oligotrophic conditions and high salinity, as in the case of the Red Sea mangroves (Almahasheer et al., 2016; Anton et al., 2020; Arshad et al., 2020; Qashqari et al., 2020; Thomson et al., 2022b). Compared to similar ecosystems e.g., in South Africa and Australia, the Red Sea mangrove sediments have a reduced particulate organic carbon content (up to 50% less) and up to a third of the particulate organic nitrogen (Thomson et al., 2015; Booth et al., 2019a). All these selection forces make Red Sea mangroves one of the most extreme coastal ecosystems on earth, possibly harboring unique and overlooked microorganisms.

We, therefore, hypothesize that such habitat acts as microbial hotspots of microbial diversity and, in particular, as reservoirs for hydrocarbon degrader bacteria not yet isolated and described. So far, many bacterial lineages still have only a few or no cultivated representatives, and only ∼23,000 species have been validly described on the List of Prokaryotic names with Standing in Nomenclature (LPSN) database (Parte et al., 2020). The reasons for failing cultivation and isolation of microorganisms in the laboratory are various (Montgomery, 2020). Some are complex, such as the need for specific growth signals (Bruns et al., 2002; Nichols et al., 2008), dependence on other microorganisms (D’Onofrio et al., 2010; He et al., 2015), development as microcolonies (Davis et al., 2011) and absence of suitable electron acceptors and/or essential nutrients (Köpke et al., 2005), while others are simpler to address, such as an insufficient incubation periods (Janssen et al., 2002; Davis et al., 2005), and the control of fast-growing species that can overcome those with slower growth (Kato et al., 2018). Several studies over the past years have shown that it is possible to isolate previously uncultivated microorganisms by using innovative strategies and approaches, such as low nutrient concentrations and longer incubation times (Janssen et al., 2002; Davis et al., 2005; Pulschen et al., 2017; Sefrji et al., 2021a,b, 2022). Moreover, cultivation success can be improved using whole metagenome analysis to unravel microorganism key metabolic pathways that can inspire new cultivation strategies (Hedlund et al., 2014; Cross et al., 2019), as well as by understanding the trophic interactions occurring within the overall microbial community (Zamkovaya et al., 2021).

Here, we aim to isolate novel previously uncultivated bacteria from the Red Sea oligotrophic arid mangrove sediments (Anton et al., 2020), unraveling the diversity of the microbial “dark matter” and rare ecosystem players (Solden et al., 2016; Jousset et al., 2017; Zamkovaya et al., 2021). To achieve our goals, we implement an extended incubation period and oligotrophic conditions combined with hydrocarbon substrates (i.e., crude oil) as C-source. With this approach, we expect to favor the growth of slow-growing hydrocarbon degrader bacteria not usually detected with standard cultivation methods. This approach reveals the vast diversity of taxonomically novel microbial hydrocarbon degraders within a collection of a few hundred isolates. Among the isolates, we describe and characterize a novel halophilic hydrocarbon-degrading bacterial species Nit1536^T^ within the genus *Nitratireductor*, for which the name *Nitratireductor thuwali* is proposed.

## Materials and methods

### Sampling, enrichment cultures, isolation and purification

Samples of sediment were collected from a fringing mangrove characterized by coarse sandy sediment, with a large part made by coral debris (Basaham et al., 2014), which stands at the King Abdullah Economic City (KAEC), Saudi Arabia (22°24′N, 39°05′E; Supplementary Figure 1). The mangrove sediment has been used as inoculum for the cultivation experiments and three different media were used: (i) ONR7a, for 1 L: NaCl 22.79 g, Na_2_SO_4_ 3.98 g, KCl 0.72 g, NaBr 83 mg, NaHCO_3_ 31 mg, H_3_BO_3_ 27 mg, NaF 2.60 mg, NH_4_Cl 0.27 g, Na_2_HPO_4_ × 7 H_2_O 89 mg, TAPSO 1.30 g, MgCl_2_ × 6 H_2_O 11.18 g, CaCl_2_ × 2 H_2_O 1.46 g, SrCl_2_ × 6 H_2_O 24 mg and, FeCl_2_ × 4H_2_O 2 mg; (ii) Mineral Salt Medium (MSM), for 1 L: Na_2_HPO_4_ 2.79 g, KH_2_PO_4_ 2 g, (NH_4_)_2_SO_4_ 1 g, Ca(NO_3_)_2_4H_2_O 0.05 g, ammonium iron(III) citrate 0.01 g and, MgSO_4_ × 7H_2_O 0.02 g; (iii) filtered Red Sea water (FSW). Liquid cultures were set up using 5 g of sediments as inoculum, 70 mL of medium, and 750 μL of Arabian Light crude oil (provided by Saudi Aramco) as the only carbon source (1% v/v). The microcosms were incubated for 20 days in aerobic conditions at 26°C, according to the temperature of the sediment at the sampling time. After the incubation time, 10-fold serial dilutions of the enriched cultures were prepared, ranging from the undiluted sample to 10^–5^; 100 μl of each dilution were then plated onto Petri dishes containing the same medium used for the liquid enrichment culture agarized with 0.8% of Noble Agar (Difco) and were incubated at 26°C for 10 days until the growth of bacterial colonies was observed. A total of 300 colonies were picked from the three media, singularly re-streaked on the respective media and further incubated; this purification step was applied three times. The colonies that showed growth (*n* = 203) were further transferred on Marine broth (MB) agar medium (BD Difco Fisher Scientific; composition: 5 g peptone, 1 g yeast extract, 0.1 g C_6_H_5_FeO_7_, 19.45 g NaCl, 5.9 g MgCl_2_, 3.24 g MgSO_4_, 1.8 g CaCl_2_, 0.55 g KCl, 0.16 g NaHCO_3_, 0.08 g KBr, 34 mg SrCl_2_, 22 mg H_3_BO_3_, 4 mg Na_2_SiO_3_, 2.4 mg NaF, 1.6 mg NH_4_NO_3_, 8 mg Na_2_HPO_4_; final salinity using a refractometer, 4%) for a faster-growth. Colonies’ purity was evaluated at the microscope before storing the strains at –80°C as a cell suspension in 25% glycerol for long-term storage.

### De-replication of bacterial isolates and identification of haplotype representatives

Genomic DNA was extracted from isolates by boiling lysis protocol (Marasco et al., 2012). A single bacterial colony was picked from the agar culture and suspended in 50 μL of sterile Tris-HCl buffer (10 mM, pH 8.0) in PCR tubes. Suspended cells were subjected to 10 min of boiling at 95°C using iCycler thermocycler (Bio-Rad) and afterward kept in ice for 10 more minutes. The samples were then centrifuged at 13,000 rpm for 10 min at 4°C. The supernatant containing DNA was used as a template for PCR amplification. First, we amplified the intergenic transcribed spacers (ITS) between the 16S and 23S rRNA genes as tool for strain typing to discriminate bacterial strains at the species and intraspecies levels (Daffonchio et al., 2003). We used the primer-set ITSf and ITSr (Boyer et al., 2001). The PCR mix was prepared in 20 μL with 0.50 U/reaction of *Taq* DNA polymerase (Thermo-Fisher Scientific), 1 × PCR buffer, 1.5 mM MgCl_2_, 0.25 mM dNTPs mix, 0.50 μM each primer (SIGMA) and 2 μL of template DNA. The PCR thermal protocol included an initial denaturation at 95°C for 5 min, followed by 30 cycles of 94°C for 45 s, 50°C for 1 min, and 72°C for 2 min, and final elongation at 72°C for 10 min. PCR products were separated by gel electrophoresis on 1.5% agarose gel, and the fingerprinting profiles of the 16S-23S rRNA ITS region were visualized using a Gel Doc System (Bio-Rad). Isolates showing the same banding pattern were grouped in haplotypes. For each 16S-23S rRNA ITS-haplotype, a representative strain was selected for further phylogenetic identification through amplification and sequencing of the 16S rRNA gene. Three PCR were conducted by using universal primer sets, which amplify three partially overlapping regions of the 16S rRNA gene: 27F/785R (fragment F1; PCR product of ≈750 bp), 341F/907R (F2; ≈550 bp) and 785F/1492R (F3; ≈700 bp). PCR mix was the following: in 50 μL of the final volume of reaction, 0.02 U/μL (corresponding to 1.0 U/reaction) of *Taq* DNA polymerase (Thermo-Fisher Scientific), 1 × PCR buffer, 1.5 mM MgCl_2_, 0.2 mM dNTPs mix, 0.3 μM each primer (SIGMA) and 1 μL of template DNA were used. The PCR thermal protocol included an initial denaturation at 94°C for 5 min, followed by 30 cycles of 94°C for 45 s, 52, 59, and 55°C (respectively, F1, F2, and F3) for 1 min, 72°C for 1 min, and final elongation at 72°C for 10 min. The sizes of the expected PCR products were evaluated on 1.5% agarose gel, then purified with Illustra ExoProStar 1 Step (GE Life-sciences) and then sequenced with both forward and reverse primer using the Sanger method (KAUST Bioscience Core Lab). Electropherograms of each sequence were checked for quality, edited, and assembled with Geneious version 8.1.9 (Biomatters) to obtain an almost full-length sequence of the 16S rRNA gene (1,300–1,450 bp). The sequences obtained were then compared through the Basic Local Alignment Search Tool (BLAST) algorithm against the reference RNA sequences database (refseq_rna) of the National Center for Biotechnology Information (NCBI). A phylogenetic tree including representative strains of each ITS haplotype (Supplementary Table 1) was performed using the Aligner tool of MEGA-X software package (Kumar et al., 2016). Using the same software, we removed the gaps to eliminate poorly aligned positions and divergent regions of the 16S rRNA gene alignment and make it more suitable for phylogenetic analysis. The phylogenetic neighbor-joining and maximum likelihood trees were constructed. A bootstrap analysis of 1,000 re-samplings was used to evaluate the tree topology (Felsenstein, 1985). The bacterial 16S rRNA gene sequences were deposited in the GeneBank database under the accession numbers ON408473–ON408537.

### Physiological, biochemical, and chemotaxonomy analysis

An internal transcribed spacer (ITS) haplotype composed of four strains, namely, Nit1531, Nit1536, Nit1537 and Nit1539, was selected for further investigation because their 16S rRNA gene sequence showed 95.19 ± 0.92% identity with the previously isolated and published *Nitratireductor pacificus* pht-3B (Lai et al., 2011a) type strains (Supplementary Table 1). Since the four strains had 16S rRNA genes similarity >99% and the same Enterobacterial Repetitive Intergenic Consensus (ERIC)-PCR profile (Supplementary Figures 2, 3), only one strain, namely, Nit1536, was further characterized and proposed as type strain (here therefore, Nit1536^T^). The standard Gram-stain protocol was followed to determine the cell wall composition, i.e., stain purple for Gram-stain-positive bacteria with thick layers of peptidoglycan (90% of cell wall) vs. stain pink for Gram-stain-negative bacteria with thin layers of peptidoglycan (10% of wall) (Madigan et al., 2008). The cell morphology of the Nit1536^T^ strain was determined by scanning electron microscopy on a Nova Nano 630 SEM (FEI) at the Imaging Core Lab at King Abdullah University of Science and Technology. Bacterial cell motility was evaluated by using the MB medium with 0.35% agar. The ability of the Nit1536^T^ strain and its closest relative *N. aquibiodomus* NL21*^T^* (DSM 15645) and *N. kimnyeongensis* Ky 101*^T^* (DSM 19185) to grow at various temperatures (10, 20, 30, 40, and 50°C) was assessed in MB medium. At the same time, salt tolerance was tested by preparing saltless MB (absence of NaCl, MgCl_2_, Na_2_SO_4_, CaCl_2_, and KCl) with the addition of different concentrations of NaCl (range, 0–19% of NaCl with an increment of 1%, w/v). The oxidase activity was tested by using oxidase test strips (Sigma-Aldrich) and the catalase by using 3% (v/v) hydrogen peroxide solution (Aladame, 1987). Indole production was tested by adding Kovac’s reagent to the bacterial culture and evaluating the color change. Nitrate reduction ability was determined using a nitrate reduction kit (Sigma-Aldrich) and following the manufacturers’ instructions. The nitrate broth medium was modified and supplemented with 4% (w/v) NaCl. Screening for amylase, protease, lipase and cellulase activities was evaluated using MB agar plates containing 1.5% starch, casein, tween 80, and cellulose as substrates, respectively, (Liu et al., 2019). The capacity to grow on linear alkanes, i.e., hexane, nonane and dodecane, and on aromatic hydrocarbons, i.e., toluene and xylene, was further evaluated by inoculating bacteria on SW plates spread with 100 μl of the listed hydrocarbons; plates without hydrocarbons were used as control. Further phenotypic characterizations of the Nit1536^T^ strain were performed with the Biolog^®^ Phenotype Microarray (PM). C-source metabolism, ions and osmolytes tolerance, and pH range were screened using PM1, PM2, PM9, PM10, PM11, and PM12 and following the manufacturing instructions. Biolog^®^ PM plates were incubated in the Omnilog incubator/reader, and the changes in color in the wells were measured every 15 min. OmniLog^®^ phenotype microarray software was used for the data analysis (Jałowiecki et al., 2017).

To evaluate the production of osmolytes by the bacterial strain, 1 mL of Nit1536^T^ culture was diluted 2 × in methanol/water (50/50), and the solution was analyzed using LC-HRMS equipped with a reversed stationary phase column and ESI source operated under + ve mode at the Analytical Chemistry Core Lab in KAUST. Briefly, 10 μL of methanol-water blank, caffeine (QC), and 10 μL of the analytes were injected separately into the LC using a SEPAX column (SFC-DIOL 150 mm × 4.6 mm packed with 5 μm particles size). The separation was performed under gradient using water with 2 mM ammonium formate, 0.1% formic acid and acetonitrile, and 0.1% FA. The flow rate was 450 μL/min. The analysis was performed using a Thermo LTQ Velos Orbitrap mass spectrometer (Thermo Scientific, Pittsburgh, PA, USA) equipped with a heated ESI ion source. The mass scan range was set to 100–2,000 m/z, with a resolving power of 100,000. The m/z calibration of the LTQ-Orbitrap analyzer was performed in the positive ESI mode using a solution containing caffeine, MRFA (met-arg-phe-ala) peptide and Ultramark 1,621 according to the manufacturer’s guidelines. The ESI was performed with a heated ion source equipped with a metal needle and operated at 4 kV. The source vaporizer temperature was adjusted to 400°C, the capillary temperature was set at 270°C, and the sheath and auxiliary gases were optimized and set to 30 and 15 arbitrary units, respectively.

For chemotaxonomic analysis, cells of the Nit1536^T^ strain and its closest relatives *N. aquibiodomus* NL21*^T^* (DSM 15645), *N. kimnyeongensis* Ky 101*^T^* (DSM 19185), *Nitratireductor* sp. HSD-9*^T^* (DSM 19383) and *Nitratireductor* sp. SL014B-25A2 (DSM 22977) were grown on MB and harvested by centrifugation at the mid-exponential phase, washed and then freeze-dried. Analysis of fatty acid, polar lipids and respiratory quinones was carried out by DSMZ Services (Leibniz-Institut DSMZ, Deutsche Sammlung von Mikroorganismen und Zellkulturen GmbH, Braunschweig, Germany).

### Sequencing, assembly and annotation of Nit1536^T^ genome

The Nit1536^T^ strain was grown aerobically and routinely maintained on MB medium. Genomic DNA was extracted from 1 mL of culture (corresponding approximately to 2 × 10^9^ cells/mL) with the Maxwell RSC Automated Nucleic Acid Purification system and the Maxwell^®^ RSC Cultured Cells DNA kit (Promega). DNA concentration was quantified using the Qubit^®^ dsDNA BR Assay Kit (Thermo-Fisher Scientific), and the quality was assessed by electrophoresis on 1% agarose gels and with a Bioanalyzer (Agilent). Sequencing of the genome of the Nit1536^T^ strain was performed at KAUST Bioscience Core Lab using one cell of the PacBio RS2 platform (Pacific Biosciences). The PacBio reads were assembled using the SMRT analysis software (PACBIO) and the HGAP.3 workflow following the hierarchical genome-assembly process (Chin et al., 2013). First, the longest reads were selected as “seed” reads, to which all other reads were mapped. Secondly, these reads were pre-assembled into highly accurate reads and used for genome assembly. Finally, a final assembly step was performed using all the reads to correct the initial assembly. The genome was annotated using Prokka (Seemann, 2014), an annotation pipeline for microbial genomes. Additional annotation and gene function prediction analysis were achieved within the Rapid Annotation using Subsystem Technology (RAST) (Aziz et al., 2008; Brettin et al., 2015), as well as the Kyoto Encyclopedia of Genes and Genomes (KEGG) (Kanehisa et al., 2016). DRAM (Shaffer et al., 2020), METABOLIC (Zhou et al., 2022), gapseq (Zimmermann et al., 2021) and anti-SMASH 5.1.2 (Blin et al., 2019) were used to infer the metabolism-related features. AMRFinderPlus (Feldgarden et al., 2021) and ResFinder (Florensa et al., 2022) were used to catalogue the antibiotic resistance genes. ABRicate^1^ and the Virulence Factor Database (Chen et al., 2016) were used to identify virulence-related features. CRISPRCasTyper (Russel et al., 2020) and DefenseFinder (Tesson et al., 2022) were used to identify CRISPRs and anti-phage defense elements. GapMind (Price et al., 2020) was run to assess the genomic completeness of amino-acid biosynthesis pathways, along with FeGenie (Garber et al., 2020) and manual searches via RAST/NCBI blastp (Johnson et al., 2008) and Pfam (Mistry et al., 2021) to identify iron-metabolism-specific features. The tools NCBI-genome-download^2^ and bit-dl-NCBI-assemblies (Lee, 2022) were used to download available representative type-species^3^ genomes belonging to the genus *Nitratireductor*. GenBank accession numbers for the whole-genome sequence of Nit1536^T^ strain is CP030941-3.

### Phylogenetic analysis of Nit1536^T^ strain

The phylogeny of the 16S rRNA gene sequences was done using the MEGA-X software as described previously with the phylogenetic neighbor-joining and maximum likelihood trees that were constructed with a bootstrap analysis of 1,000 re-samplings to evaluate the tree topology (Felsenstein, 1985). We also used the GTDB-Tk software (Chaumeil et al., 2019) to build the phylogenetic tree of multi-locus sequence analysis (MLSA) using 120 concatenated single-copy proteins obtained from the genome of the Nit1536^T^ strain and other close type species belonging to the *Oricola*, *Chelativorans*, *Nitratireductor* and a subset of *Mesorhizobium* genera. The genome sequence of the Nit1536^T^ strain and those of other species belonging to the *Nitratireductor* genus were imported into the JSpeciesWS, an online service for *in silico* calculation of the extent of identity between two genomes. The online software measures the average nucleotide identity (ANI) based on BLAST (ANIb), as well as correlation indexes of tetra-nucleotide signatures (Tetra) (Burall et al., 2016; Richter et al., 2016). We further used the Genome-to-Genome Distance Calculator (GGDC) online server and the EzAAI software to calculate *in silico* the estimated value of DNA-DNA hybridization (DDH) and the average amino acid identity (AAI) of Nit1536^T^ strain with the closest relative species, respectively, (Meier-Kolthoff et al., 2013; Kim et al., 2021).

## Results

### Phylogenetic diversity of the cultivable hydrocarbon-degrading bacteria from arid mangrove sediments

**FIGURE 1:**
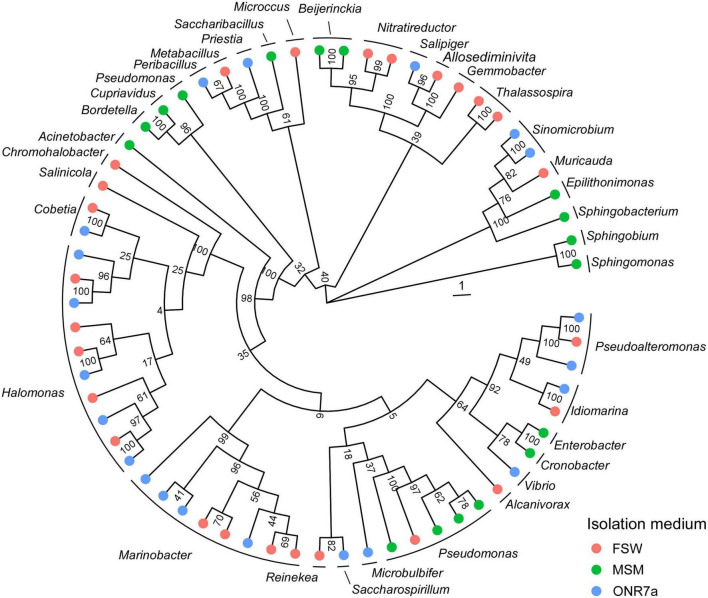
Phylogenetic tree of cultivable bacteria harbored by mangrove oligotrophic sediments. The colors on the outer ring refer to the medium used for the isolation, namely, FSW (red), MSM (green), and ONR7a (blue). A representative sequence for each ITS haplotype is included (see Supplementary Table 1 for the full list).

From the arid mangrove sediments of King Abdullah Economic City (KAEC, Thuwal), we established a collection of hydrocarbon-degrading bacteria capable of growth on the Arabian light crude oil as carbon sources by using three different media, namely, MSM, ONR7a and FSW (Supplementary Table 1). We obtained a total of CFU per g that ranged between 1.07 × 10^5^ on ONR7a and 3.98 × 10^8^ on MSM (Supplementary Table 2). We successfully isolated 55, 60 and 88 bacterial strains from MSM, ONR7a and FSW plates, respectively. These bacteria were further sub-grouped in 17, 21, and 26 based on their ITS fingerprinting (Supplementary Table 1). All three media favored the isolation of bacteria belonging to the Proteobacteria phylum (94.5, 88.3, and 92.0% in MSM, ONR7a and FSW, respectively; Figure 1 and Supplementary Table 1). On MSM medium, 36.4% of the isolates belonged to the *Alphaproteobacteria*, 7.3% to *Betaproteobacteria* and 50.9% to *Gammaproteobacteria*, while on ONR7a and FSW the *Gammaproteobacteria* (86.7 and 71.6%, respectively) were largely dominant (Figure 1). The selection driven by the medium was more evident at the genus level; 35 different genera were detected, and no one was common among the three media (Figure 1 and Supplementary Figure 4). *Pseudomonas* and *Sphingobium* (30.9 and 27.2%, respectively) were the dominant genera within the MSM collection, while *Marinobacter*, *Pseudoalteromonas*, and *Halomonas* (36.7, 20, and 16.7%, respectively) those in the ONR7a collection, and *Halomonas*, *Alcanivorax*, and *Cobetia* (20.4, 14.7, and 13.6%, respectively) in the FSW collection (Supplementary Figure 4). Along with these genera frequently cultivated, we also identified bacteria belonging to “rare” (or uncommon) genera, i.e., not often isolated and with few representatives sequenced (Pedrós-Alió, 2012). For example, we cultivated members of *Thalassospira* (at the time of writing, 12 validly described representatives reported on the LPSN website),^4^ *Nitratireductor* (10), *Pelagibaca* (synonym *Salipiger*, 9), *Roseivivax* (7), *Cobetia* (5), *Reinekea* (5), *Sinomicrobium* (5), and *Joostella* (1). These groups were present in different percentages, with the highest number of rare taxa obtained from the FSW and ONR7a media (6 and 4 genera, respectively), while no one from the MSM medium (Supplementary Figure 3).

### Phylogenetic analysis and identification of the Nit1536^T^ strain

**FIGURE 2:**
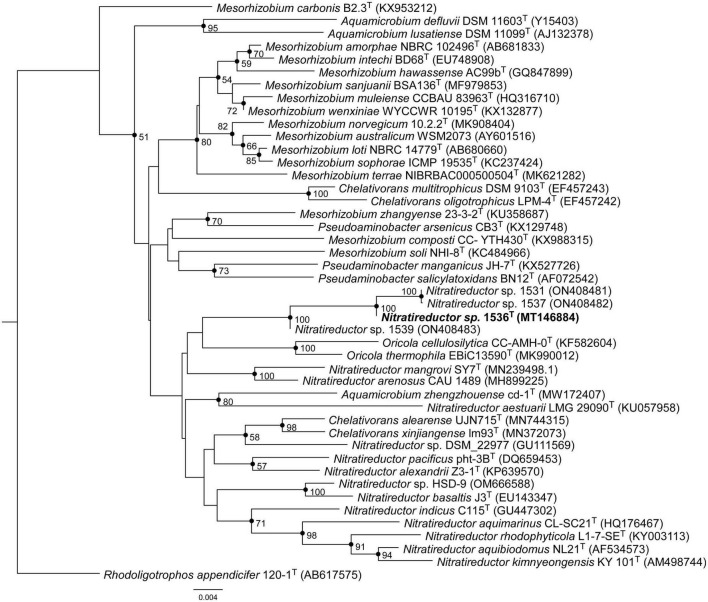
Neighbor-joining phylogenetic tree based on the bacterial 16S rRNA gene sequences showing the position of Nit1536^T^. Only bootstrap values (expressed as percentages of 1,000 replications) of >50% are shown at the branching points. Filled circles indicate branches that were also recovered using the maximum-likelihood method. *Rhodoligotrophos appendicifer* 120-1^T^ (AB617575) was used as an outgroup. Bar, 0.004 substitutions per nucleotide position.

**FIGURE 3:**
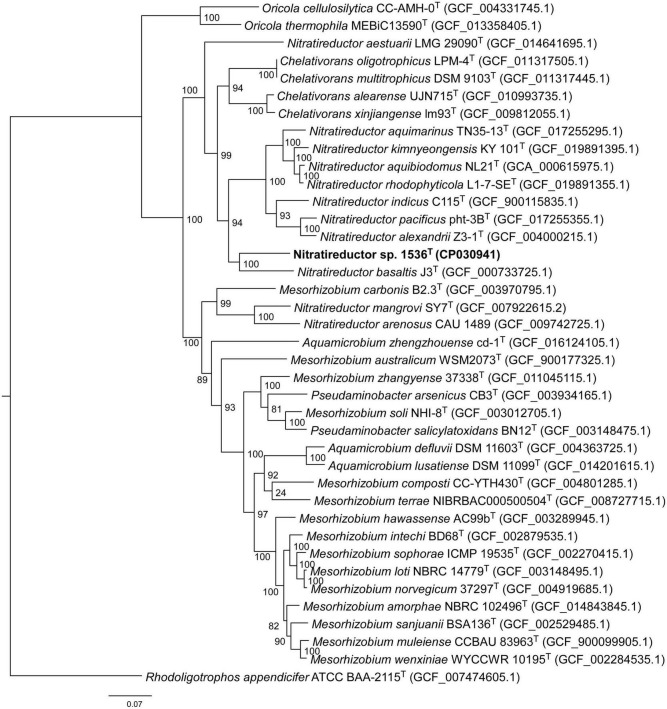
Neighbor-joining phylogenomic tree using an MLSA concatenating 120 essential single-copy genes, highlighting the position of the Nit1536^T^ strain relative to other closely related bacterial taxa within the *Phyllobacteriaceae* family. The tree was reconstructed using the software GTDB-Tk (Chaumeil et al., 2019). Numbers at the nodes designate bootstrap support values resulting from 1,000 bootstrap replicates. Bar, 0.07 substitution per nucleotide position. *Rhodoligotrophos appendicifer* ATCC BAA-2115T (GCF_007474605.1) was used as an outgroup.

Comparative analysis of 16S rRNA gene sequences showed that one haplotype was related most closely to *Nitratireductor pacificus* pht-3B^T^ and *Nitratireductor indicus* C115^T^ with 95.41 and 95.36% similarity, respectively. The phylogenetic tree revealed that Nit1536^T^ formed a distinct phylogenetic lineage from all the other species within the *Nitratireductor* genus (Figure 2). The sequence similarity calculations based on evolutionary distance demonstrated that the Nit1536^T^ strain represents a putative novel species. Multi-locus sequence analysis (MLSA) was performed against close type species belonging to the *Oricola*, *Chelativorans*, *Nitratireductor*, and a subset of *Mesorhizobium* genera, confirming the separation of the Nit1536^T^ strain from the other species within the *Nitratireductor* genus (Figure 3). Average nucleotide identity based on BLAST (ANIb) revealed that Nit1536^T^ had a percentage of identity between 73.03 and 75.29% with the other closely related species (Table 1), values that are below the 96% species-embracing threshold (Richter et al., 2016). Similarly, *in silico* DNA-DNA hybridization (DDH) showed values ranging between 19 and 21% (Table 1) that are significantly lower than 70% used as a cut-off value to delineate a new bacterial species (Meier-Kolthoff et al., 2013). Furthermore, the average amino acid identity (AAI) analysis showed that the similarity between our strain and all the other closest species ranges between 69.6 and 74.5% (species delimitation threshold, 95% (Konstantinidis et al., 2017; Table 1). The results of the phylogenetic analyses and genome relatedness suggested that the Nit1536^T^ strain represents a novel species in the genus *Nitratireductor*, for which the name *Nitratireductor thuwali* sp. nov. is proposed.

**TABLE 1:** (i) Average nucleotide identity via BLAST (ANIb) and percentage of aligned sequences in brackets, (ii) *in silico* DNA-DNA hybridization (DDH) calculation, and (iii) average amino acid identity (AAI) of Nit1536^T^ against other representatives of the *Nitratireductor* genus, namely, *N. aestuarii* 2-2-12-1^T^ (Ou et al., 2017), *N. alexandrii* Z3-1^T^ (Jiang et al., 2020), *N. aquibiodomus* NL21^T^ (Labbé et al., 2004), *N. aquimarinus* TN35-13 (non-type strain), *N. basaltis* J3^T^ (Kim et al., 2009), *N. indicus* C115^T^ (Lai et al., 2011b,^2012^), *N. kimnyeongensis* Ky 101^T^ (Kang et al., 2009), *N. pacificus* pht-3B^T^ (Lai et al., 2011a), *N. rhodophyticola* L1-7-SE^T^ (Kim et al., 2022), and *N. mangrovi* SY7^T^ (Ye et al., 2020).

Reference genome	Accession number	ANIb (%)	DDH (%)	AAI (%)
*N. aestuarii* 2-2-12-1^T^	GCF_014641695.1	71.9 (35.2)	19	69.6
*N. alexandrii* Z3-1^T^	GCF_004000215.1	73.3 (41.4)	19.7	69.8
*N. aquibiodomus* NL21^T^	GCF_000615975.1	74.2 (47.1)	20.3	74
*N. aquimarinus* TN35-13*	GCF_017255295.1	74.5 (46.1)	20.7	73.7
*N. basaltis* J3^T^	GCF_000733725.1	73.7 (45.9)	19.7	74.5
*N. indicus* C115^T^	GCF_000300515.1	73.4 (43.8)	20.2	73
*N. kimnyeongensis* Ky 101^T^	GCF_019891395.1	73.2 (46.9)	19.5	73.9
*N. pacificus* pht-3B^T^	GCF_000300335.1	75.6 (47.8)	21	74.1
*N. rhodophyticola* L1-7-SE^T^	GCF_019891355.1	74.1 (46.4)	20.1	62.4
*N. mangrovi* SY7^T^	GCA_007922615.2	73.2 (39.4)	19.7	57.0

*Non-type strain; we used TN35-13 strain because its 16S rRNA gene had 99.79% similarity with the one of the type strain CL-SC21^T^ (Jang et al., 2011) for which genome was not available.

### Morphological, physiological, and biochemical characterization of Nit1536^T^

**FIGURE 4:**
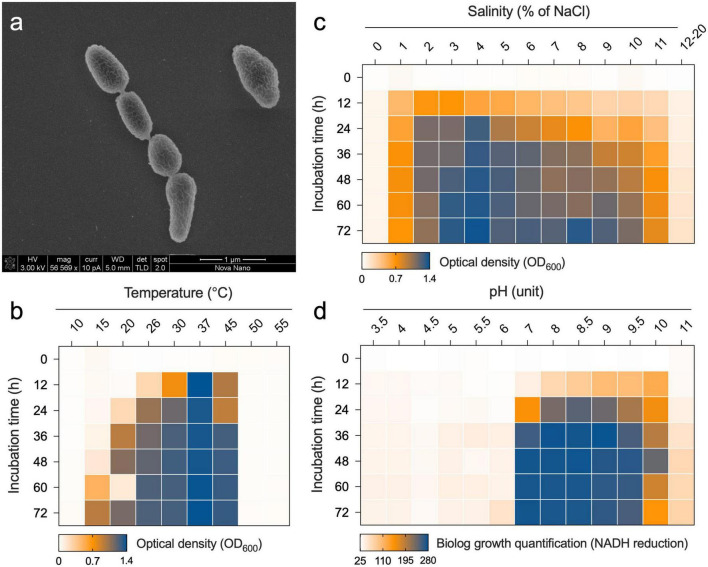
**(a)** Transmission electron micrographs of Nit1536^T^. The bar length is 1 μm. Growth of Nit1536^T^ at different **(b)** temperatures and **(c)** salinities (% of NaCl) is reported as the average value of OD_600_ (*n* = 3). **(d)** Metabolism of Nit1536^T^ under different pH is analyzed using Biolog PM and reported as the quantity of dye formation.

The Nit1536^T^ strain is a short rod-shaped, Gram-negative, aerobic, and non-motile bacterium. Although several genes for flagellar synthesis are found, the genes encoding for chaperone proteins FlgN, FliS, FliT, the filament cap FliD, regulatory proteins FlhC and FlhD and the biosynthesis proteins FliO and FliH are not present in the genome (Supplementary Result 1). One cell is typically 0.9 μm in length and 0.4 μm in width (Figure 4a). Colonies are circular with 1–2 mm in diameter and regular edges, smooth, shiny, creamy in colour, possibly due to the absence of biosynthetic genes involved in the production of pigments (Supplementary Result 1). The Nit1536^T^ strain is a mesophilic bacterium able to grow between 15 and 45°C (optimum 37°C; Figure 4b). It is able to grow in a medium containing 1–14% of NaCl (optimum 4%; Figure 4c), making it a moderately halophilic bacterium (Zhu et al., 2007) adapted to the salinity conditions of the Red Sea mangroves [up to 15% (Fusi et al., 2022)] where no fresh water inputs and low-tidal range occur in summer due to El Niño–Southern Oscillation (ENSO). The Nit1536^T^ strain also has metabolic activity in the presence of various osmolytes and ions, such as betaine, sarcosine, ectoine, choline, proline, trehalose, ethylene glycol, and others (total of 27 compounds; full list in Supplementary Table 4), while a full metabolic inhibition occur in the presence of potassium chloride (5 and 6%), sodium formate (1–6%), sodium lactate (1–12%), sodium benzoate (20–200 mM), ammonium sulphate (10–100 mM) and sodium nitrite (20–100 mM). The Nit1536^T^ strain shows similar metabolic activity at pH values between 7 and 10, with an optimum at pH 8 (Figure 4d). These findings are in accordance with its environment of origin, where the pH of the sediments varied from 7.5 to 10 over the course of the year (Fusi et al., 2022). Under the optimal conditions (i.e., 37°C, 4% NaCl and pH 8), the doubling time for growth is 6.5 h, confirming its classification as a slow-growing bacterium.

Of the 190 carbon sources available in the PM1 and PM2 microplates, the Nit1536^T^ strain shows an active metabolism on 57 carbon sources, such as, among others, L-histidine, dihydroxy acetone, arbutin, 2-deoxy-D-ribose, 5-keto-D-gluconic acid, L-lyxose L-seryne, D-glucosamine and oxalomalic acid, that induced a higher metabolic rate (Supplementary Figure 5). In addition, besides its growth on crude oil, when single hydrocarbons are considered, the Nit1536^T^ strain grows on linear alkanes, such as hexane, nonane and dodecane, but not on aromatic hydrocarbons (toluene and xylene). The metabolism of Nit1536^T^ strains is inhibited at all four antibiotic concentrations present in Biolog^®^ PM plates for four antibiotics (novobiocin, spectinomycin, nafcillin, and spiramycin) and two inhibitor compounds (benzethonium chloride and dodecyltrimethylammonium bromide). In contrast, 29 antibiotic/inhibitor compounds did not affect the bacterial metabolism (i.e., active metabolism at all concentrations of the antibiotic), and 11 only partially affected it (i.e., active metabolism only at low concentrations of the antibiotic) (details in Supplementary Table 5). The antibiotic resistance ability of this strain was confirmed by the AMRFinderPlus tool, which revealed >80% query coverage and 56–64% identity at the amino-acid level to class A beta-lactamases (bla) and Fosfomycin resistance hydrolase (fosX). It is important to note that since antibiotic resistance is a strain-specific behavior, we can attribute this antibiotic resistance/sensitivity profile only to this strain.

The Nit1536^T^ isolate is positive for oxidase and catalase, while it was negative for indole production, nitrate reduction, amylase, protease, lipase and cellulase activities. Chemotaxonomic analysis indicated that the predominant respiratory ubiquinone is Q-10, as observed in the other *Nitratireductor* genus species (range, 96.6–98.4%) (Chen et al., 2015) and many members of the *Alphaproteobacteria* class (Fukuda et al., 2012). The major fatty acids (>5%) of the Nit1536^T^ strain are a combination of C_18:1_ *_ω_* 7*c* and/or C_18:1_ *_ω_* 6*c* [summed feature 8 (SF8), 66.54%], C_19:0_ cyclo *_ω_* 8*c* (14.31%) and C_18:0_ (8.62%). Its fatty acid composition is more similar to those of *N. aquibiodomus* NL21^T^ and *N. kimnyeongensis* Ky 101^T^ than those of *Nitratireductor* spp. HSD-9^T^ and SL014B-25A2. However, the different relative abundance of its saturated straight chain fatty acids (C_16:0_, C_17:0_, C_18:0_, and iso-C_17:0_) shows the unicity of the Nit1536^T^ strain (Table 2). As for its closest related species, the Nit1536^T^ strain has phosphatidylcholine (PC), phosphatidylglycerol (PG), phosphatidylethanolamine (PE), diphosphatidylglycerol (DPG), and glycolipid (GL) as dominant polar lipids (Supplementary Figure 6). It also shows that phosphatidyl-methyl ethanolamine (PME) and several unidentified lipids (L) are not present in the other strains.

**TABLE 2:** Cellular fatty acid composition (%) of strain Nit1536^T^ and its closest related species *Nitratireductor aquibiodomus* NL21^T^ (DSM 15645, from marine denitrification system) (Labbé et al., 2004), *N. kimnyeongensis* Ky 101^T^ (DSM 19185, from dried seaweed) (Kang et al., 2009), *Nitratireductor* sp. HSD-9^T^ (DSM 19383, from beach sand) and *Nitratireductor* sp. SL014B-25A2 (DSM 22977 non-type strain, from oil-polluted soil).

Fatty acid type	Fatty acid composition	Nit1536^T^	NL21^T^	Ky 101^T^	HSD-9^T^	SL014B^#^
Saturated straight chain	C_16:0_	2.40	0.74	0.67	**6**.**56**	3.03
	C_17:0_	0.67	0.74	2.49	**14**.**20**	4.10
	C_18:0_	**8**.**62**	2.10	3.12	**15**.**65**	**5**.**97**
Saturated branch chain	iso-C_17:0_	2.87	2.79	1.81	**7**.**41**	**11**.**74**
Unsaturated branch chain	C_20:1_*_ω_* 7*c*	0.71	0.76	1.26	1.28	0.17
	C_20:2_*_ω_* 6*c*9	0.75	0.30	0.17	0.28	0.86
Hydroxylated	iso-C_15:0_ 3-OH	0.97	1.56	0.62	3.04	-
	C_18:0_ 3-OH	0.49	0.63	0.18	0.63	0.13
Cyclopropane acids	C_19:0_ cyclo *_ω_* 8*c*	**14**.**31**	**14**.**49**	**20**.**97**	**22**.**97**	**25**.**81**
Methyl	11 methyl-C_18:1_*_ω_* 7*c*	1.13	0.21	1.24	0.31	0.88
Other	SF3*	0.54	0.38	0.31	**5**.**25**	0.25
	SF8*	**66**.**54**	**73**.**94**	**63**.**60**	-	**40**.**88**

^#^SL014B-25A2 is available at DSMZ but it is not a type strain. *Summed features are groups of two or three fatty acids that could not be separated by GLC with the MIDI system; SF3 (summed feature 3) comprises iso-C_15:0_ 2-OH and/or C_16:1ω_ 7c and/or C_16:1ω_ 6c, while summed feature 8 (SF8) comprises C_18:1ω_ 7c and/or C_18:1ω_ 6c. Values are shown as percentages of total fatty acids. The prevalent fatty acid components (>5%) in the different bacterial strains are indicated in bold; –, not detected. Fatty acids accounting for less than 1% in all the strains are not reported. The data reported were performed by DSMZ in this studied.

Morphological, physiological and chemo-taxonomic characteristics of the Nit1536^T^ strain are compared to those of its closest relative type strains, namely, *N. aquibiodomus* NL21^T^ and *N. kimnyeongensis* Ky 101^T^ (Tables 2, 3), as well as with non-yet recognized species *Nitratireductor* sp. in the case of chemo-taxonomic (Table 2). These results corroborate the unicity of this strain previously suggested by the 16S rRNA gene sequence analysis and *in silico* DNA–DNA hybridization studies (Figures 2, 3 and Table 1). Based on these, we conclude that strain Nit1536^T^ represents a novel species within the genus *Nitratireductor*, for which the name *Nitratireductor thuwali* sp. nov. is proposed.

**TABLE 3:** Differential phenotypic characteristics of strain Nit1536^T^ and closely related species of *Nitratireductor* genus, namely, *N. aquibiodomus* NL21^T^ (DSM 15645) (Labbé et al., 2004) and *N. kimnyeongensis* Ky 101^T^ (DSM 19185) (Kang et al., 2009).

Characteristic	Nit1536^T^	NL21^T^	Ky 101^T^
Isolation source	Sediment of arid mangrove	Marine denitrification system	Dried seaweed
**Morphology**			
Cell shape	Short rods	Rods	Rods
Cell size (μm)	0.9 × 0.4	2.5 × 1.0	1.8 × 0.4
Pigment production	–	–	+
**Physiology**			
Motility	–	+	+
Temperature (°C) range for growth	15–45	20–40	20–40
Temperature (°C) optimum	37	30	30
NaCl (%) range for growth	1–14	1–7	0–7
NaCl (%) optimum	4	3	3
pH range for growth	6–10	6–10	6–12
pH optimum	8	7	8
**Genome** *			
DNA G + C content (mol%)	63.9	57	60.4
**Enzymatic activity**			
Indole production	–	–	–
Nitrate reduction	–	+	+
Oxidase	+	+	+
Catalase	+	+	+
Amylase	–	–	–
Lipase	–	–	–

Characteristics are scored as (+) present and (–) absent.

*Data obtained from published genome.

### Metabolism-related features inferred from Nit1536^T^ genome

The genome of the proposed new bacterial species *Nitratireductor thuwali* Nit1536^T^ presents three replicons consisting of one main circular chromosome consisting of 4,216,528 bp in length (Supplementary Figure 7) and two plasmids consisting of 317,553 (p1536_1) and 212,699 bp (p1536_2) in length, respectively. We observed 4,558 CDS, 58 RNAs, and a G + C of 63.9 mol%. Two identical copies of a full-length 16S rRNA gene (1,544) have been detected, one in the chromosome and one in one of the two plasmids (p1536_1), which, since it was harboring a housekeeping gene, cannot be strictly defined as a plasmid (Tazzyman and Bonhoeffer, 2014).

*Respiration.* Analysis of the genome reveals that all subunits of cytochrome bc1 complex and NADH: quinone oxidoreductase complex were encoded, confirming the aerobic respiration in this bacterium. Components of F-type ATPase (complex V) are also predicted in the genome. The two core subunits (CydA and CydB) of cytochrome bd oxidase but not the associated subunits are encoded on the genome. If the high-affinity cytochrome bd oxidase is functional in the isolate, it would not only facilitate the survival of this strain under oxygen-poor conditions (microaerophilic) typical of mangrove water-logged sediments (Sarker et al., 2021), but would also confer enhanced tolerance to nitrosative stress (NO and O_2_^–^), resistance to hydrogen peroxide, suppression of extracellular superoxide production and the ability to defend against antibacterial agents (Subedi et al., 2022).

**FIGURE 5:**
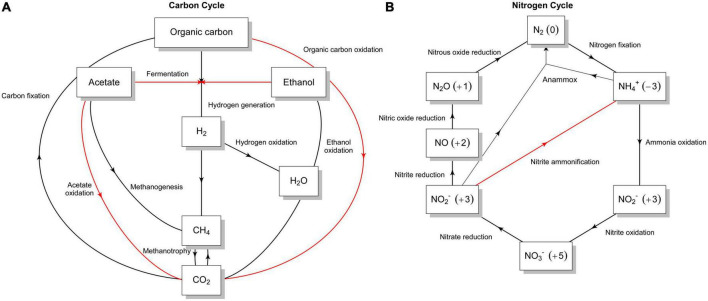
Graphical representation of different sub-pathways of **(A)** carbon and **(B)** nitrogen metabolism in the Nit1536^T^ strain. The presence of the different sub-pathways is indicated with red arrows, while their absence is indicated with black arrows. The flow graphs were created using a modified script from METABOLIC (Zhou et al., 2022).

*C-metabolism and energy pathways.* A complete tricarboxylic acid (TCA) cycle is encoded, implying that organic carbon compounds, such as carbohydrates, organic acids and polycyclic aromatic hydrocarbons, can be used as carbon and energy sources (overview in Figure 5A and Supplementary Result 1). For instance, we detected genes encoding the degradation of polycyclic aromatic hydrocarbon (PAH), including extradiol dioxygenases and rieske non-heme iron oxygenase (Supplementary Table 3A) that catalyze ring-cleavage reactions and a wide variety of mechanisms in the biodegradation of xenobiotics, respectively, (Barry and Challis, 2013). In addition, the metabolic pathways for sugar utilization were encoded in the genome such as glycolysis (EMP), Entner-Doudoroff (EDD) and the pentose-phosphate pathways, confirming the versatile metabolism observed in the presence of certain sugars with Biolog PM plates (Supplementary Figure 5). Growth on acetate or C_2_-compounds as a sole source of carbon and energy is also possible since the genome encoded acetyl-CoA synthetase, acetate transporter (*ActP*), and enzymes from the glyoxylate shunt (red arrows in Figure 5A and Supplementary Result 1). The latter pathway, along with the essential genes encoding enzymes of the beta-oxidation pathway, could confer Nit1536^T^ the ability to grow on fatty acids. Notably, the presence of formate dehydrogenases and components of the serine cycle may imply that the strain possesses the capacity to detoxify and utilize formate as a source of carbon and/or energy. Anaplerotic CO_2_ fixation for replenishment of TCA cycle intermediates could be mediated through the encoded pyruvate carboxylase or phosphoenolpyruvate carboxykinase. In contrast, autotrophic CO_2_ fixation capacity was not detected (black arrows in Figure 5A and Supplementary Result 1). No phototrophic modules are present, and no lithotrophic modules were detected for energy generation via ammonia, hydrogen, and sulphur (Supplementary Result 1). Despite the methane abundance in the mangrove sediments (Bižić et al., 2020), no pathways for methanogenesis or methanotrophy were found (black arrows in Figure 5A). Moreover, as also revealed by Biolog (Supplementary Figure 5), glycerol can serve as a sole C and energy source for Nit1536^T^ by the action of the enzyme glycerol kinase, glycerol-3-P dehydrogenase and glycerol-3-phosphate. Apart from alcohol dehydrogenases and putative lactate dehydrogenases, fermentative pathways are absent. All potential CAZymes predicted by the DRAM tool are listed in Supplementary Table 3B. For instance, the cellulose degradation pathway was predicted to be only 66% complete on the gapseq tool; hence, the isolate lacks this ability. Similarly, the chitin, lignin, pectin, starch, and mucin degradation pathways are incomplete. These results confirm the data obtained by the *in vitro* tests.

*Nitrogen metabolism and transporters.* Only genes encoding the small and large subunits of a putative assimilatory nitrite reductase (*nirBD*) and the assimilatory nitrate reductase (*nas*) were predicted in the genome (Figure 5B). Genes involved in nitrogen fixation, denitrification, dissimilatory nitrite reduction to ammonia, nitrate and nitrite reduction, and anaerobic/aerobic ammonia oxidation are absent (black arrows in Figure 5B), while those involved in urea and ammonium uptake/assimilation are encoded (Supplementary Result 1). The genome also reveals the presence of several transporters, among others those for peptides/amino acids, urea, ammonium, VitB12, sugars and organic substrates (Supplementary Result 1).

*Environmental adaptation and stress tolerance.* We further investigated the Nit1536^T^ genomic adaptation to mangrove abiotic stresses (i.e., salinity and temperature fluctuation). The Nit1536^T^ genome encodes genes pertaining to DNA repair components, such as *uvrABC*, *recAFORQJ*, uracil-DNA glycosylases (*udg*), *mutS*, *mutL*, endonucleases (*end*), and ligases (*lig*), as well as it possesses several genes responsible for the osmotic stress response (Supplementary Result 1). For instance, the genome displayed the presence of a gene cluster responsible for the biosynthesis (and/or uptake) of ectoine, glycine betaine, proline, and choline. Using HPLC high-resolution mass spectrometry technique (HPLC-HR-MS) we confirmed the production of ectoine, betaine, glutamate, proline, trehalose, alanine, glycerophosphorylcholine, and dymethylsulfoniopropionate (Supplementary Figure 8). Key genes for the synthesis of glycerol, trehalose and glutamate are also present, while those involved in the synthesis of sorbitol and sucrose are absent (Supplementary Result 1). Such compatible solutes may also act as abiotic stress protectors for plants, as shown by the Nit1536^T^ inoculation of barley plantlets grown using seawater (Supplementary Methods 1). Compared to the negative control (i.e., plant exposed to salt stress but not treated with the bacterium), the treatment with Nit1536^T^ strain promotes the fresh and dry weight of root biomass with an increasing weight of 42.9 and 7.6%, respectively, (Supplementary Table 6). It is important to note that this strain also has the genetic capacity to synthesize storage granules, such as polyhydroxyalkanoate (PHA), to potentially resist starvation, exposure to radiation, desiccation, high salinity and oxidants typical of mangrove sediments (Zhu et al., 2018; Fusi et al., 2022). This genetic capacity to synthesize PHA was also detected in the other available genomes within the *Nitratireductor* genus.

## Discussion

Molecular ecology surveys investigating the microbial communities of many different environments have revealed an impressive diversity of microbes in nature and have highlighted our inability to cultivate most of them in the laboratory (Kaeberlein, 2002; Bodor et al., 2020). However, a deeper knowledge of the metabolic features of these uncultivated but ecologically relevant microbial taxa is a fundamental step to disentangle their role in the multifunctionality of ecosystems (e.g., biogeochemical cycles), as well as their adaptation to environmental drivers. In this context, improving our ability to grow uncultivable microbes in laboratory conditions can support progresses in this challenging task. Here, alternative cultivation strategies—successfully used in the past to expand the collection of environmentally relevant but poorly represented microbial strains (Janssen et al., 2002; Rappé et al., 2002; Joseph et al., 2003; Lewis et al., 2010; Pulschen et al., 2017)—have been applied to untap microbial diversity of the arid Red Sea mangrove sediment, with particular focus on the hydrocarbon degrader bacteria.

By using oligotrophic media, hydrocarbon (crude oil) as a C-source and long incubation time, we succeed in the cultivation and isolation of slow-growing bacteria belonging to rarely cultivable (or uncommon) species and poorly described taxa, including members of *Citreicella, Nitratireductor, Joostella*, *Pelagibaca*, *Reinekea*, and *Sinomicrobium* genera. These lineages, so far, have few cultivated representatives. For instance, *Joostella* and *Sinomicrobium* genera have few validly described representatives obtained from marine/saline ecosystems but not from oil hydrocarbon-degrading communities. They are characterized by interesting features with biotechnological potentials, such as algicidal activity against toxic dinoflagellates or the production of enzymes of industrial interest (Cheng et al., 2014; Yang et al., 2014). Other genera, such as *Nitratireductor* (10 validly described species), *Citreicella* (4), *Reinekea* (4) and *Pelagibaca* (2) have been previously obtained from bacterial consortia associated with oil hydrocarbon contamination and biodegradation (Lai et al., 2012; Pan et al., 2014; Wang et al., 2014; Liu et al., 2017) or by applying oligotrophic cultivation conditions (Hahnke et al., 2015). These data point out the usefulness of low nutritional media (i.e., ONR7a and FSW), hydrocarbon C-sources and long incubation periods to favor the isolation of rare genera and slow-growing bacterial strains.

Within highly diverse ecosystems, such as mangroves, most of the bacterial taxa are in low abundance as members of the rare biosphere (Pedrós-Alió, 2012; Coveley et al., 2015). Rare taxa are mainly “tolerant strategists,” and thus can persist under limited resources and suboptimal abiotic conditions (Malik et al., 2020). Moreover, when these taxa are phylogenetic distant from the dominant/abundant taxa (e.g., phylogenetic novelty), they can act as a “backup system” able to promptly respond to environmental fluctuations and disturbance, vicariate multiple metabolic capabilities and ecosystem functions (Jousset et al., 2017; Shu et al., 2021; He et al., 2022).

In our work, within a collection of a few hundred isolates, we mainly identified strains closely related to previously described species (>97% 16S rRNA gene sequence identity) and one new species, Nit1536^T^ strain, affiliated to the genus *Nitratireductor*. The isolation and description of a novel strain belonging to a bacterial group poorly characterized but consistently detected in the environment, such as *Nitratireductor* in mangroves (Booth et al., 2019b; Li et al., 2020; Baskaran and Prabavathy, 2022; Fusi et al., 2022), represent an opportunity to better understand (i) the microbial adaptation strategies adopted to survive in the extreme and stressful conditions of such environment and (ii) their ecological/functional role in the sediment microbial community and mangrove ecosystem. According to LPSN (see text footnote 4), at the time of writing, only one *Nitratireductor* species was obtained from a mangrove, i.e., *N. mangrovi* (Ye et al., 2020), even if its nomenclature status is not yet validly published.^5^

Arid mangroves are exposed to strong selection forces, including high-temperature and salinity variation, heterogeneous nutrient availability and sharp gradient of oxygen availability (Kathiresan and Bingham, 2001), making them an “extreme” habitat (Helfer and Hassenrück, 2021; Palit et al., 2022). Microbial communities develop different strategies to adjust the cell osmotic homeostasis (Xu et al., 2018) in environments with elevated osmolarity (high salinity and dryness), including the less-expensive biosynthesis of osmoprotective compounds/compatible solutes, i.e., molecules with low molecular mass, that do not require structural modifications of the cells (Sleator, 2001). The biosynthesis of these compounds is controlled by environmental cues, such as high salinity and heat stress (Ono et al., 1998; Xu et al., 2018), and their accumulation in high concentrations ensures the physiological functions of the cell. Interesting, these compounds could also be responsible for the promoting/protecting activity mediated by Nit1536^T^ in plants (Supplementary Table 6), thus making this halophytic strain a candidate probiotic for mangrove restoration strategies and sustainable agricultural production in salt-affected soils (Soldan et al., 2019; Prittesh et al., 2020). The Nit1536^T^ strain also shows an important adaptation to manage reactive oxygen species that are typically associated with high salinity environment (Hassan et al., 2020). This trait can be important, especially in the Red Sea mangrove, where the seasonal sea level variation, i.e., tidal range and the lack of water input leave the sediment dry with massive salt accumulation (Fusi et al., 2022). Such variation in salinity strongly selects the microbial community living there (Thomson et al., 2022a). Combined with the production of compatible solutes, the Nit1536^T^ strain survives under such conditions by controlling the oxidative stresses triggered by the periodical high salinity.

The Nit1536^T^ strain shows additional peculiar adaptation to the mangrove environment because it can manage different levels of oxygen availability in the sediment dictated by the diel and seasonal tide inundation, as well as high plasticity of carbon source retrieval from the different compounds that can be available in the mangrove sediment, including hydrocarbon sources from anthropogenic activity, such as ships and oil tanker traffic, industrial and solid waste, plastic debris, and natural vents and seeps (Maiti and Chowdhury, 2013; Nagi and Abubakr, 2013; Al-Ali et al., 2015; Martin et al., 2019a). On the contrary, recalcitrant carbon (e.g., lignin) cannot be processed by this *Nitratireductor* strain and therefore it is excluded by those process of carbon degradation that involves litter/wood consumption. Considering the available nutrient source in mangrove sediment, the Nit1536^T^ strain showed the capability to assimilate ammonium and urea as nitrogen sources. Arid mangroves are N-deficient (Almahasheer et al., 2016; Anton et al., 2020; Fusi et al., 2022) and a strong adaptation for nitrogen acquisition is a necessity to survive. The Nit1536^T^ strain has the potential to use the ammonia secreted by the vast majority of marine organisms living in the mangrove stand (Wright, 1995). Exceptionally, some of these organisms, such as the killifish *Rivulus marmoratus* (Frick and Wright, 2002), are capable of producing urea that can be used by the strain, revealing its high plasticity in retrieving the nitrogen from the mangrove sediments. The capability to retain nitrogen from ammonium and urea can be an important ecosystem service mediated by Nit1536^T^ strain: it can help mangrove to recycle the nutrient avoiding out-welling by tidal export (Lee, 1995; Walton et al., 2014). Since nitrogen is one of the limiting factors for plant growth in arid mangrove (Almahasheer et al., 2016), bacteria that can recycle and retain the nitrogen in the system can be important therefore for plant growth and ultimately the overall system homeostasis.

### Description of *Nitratireductor thuwali* sp. nov.

*Nitratireductor thuwali* sp. nov. (thu.wal. ’i. L.fem.gen. n. *thuwali*, pertaining to Thuwal village, Makkah region, Saudi Arabia). Cells are Gram-stain-negative, non-motile and short rods of approximately 0.4 μm in width and 0.9 μm in length. Colonies on solid MB plates are cream, smooth, shiny, and circular 1–2 mm in diameter. Optimal growth occurs at 37°C, pH 8 and 4% NaCl. Oxidase and catalase are positive, while indole production, nitrate reduction, amylase, protease, lipase and cellulase activities are negative. Linear hydrocarbons, L-histidine, dihydroxy acetone, arbutin, 2-deoxy-D-ribose, 5-keto-D-gluconic acid, L-lyxose L-seryne, D-glucosamine and oxalomalic acid can be utilized as a sole carbon and energy source. The dominant polar lipids are phosphatidylcholine, phosphatidylglycerol, phosphatidylethanolamine, diphosphatidylglycerol, and glycolipid, along with phosphatidyl-methyl ethanolamine and several unidentified lipids. The major fatty acids (>5%) were summed feature 8 (C_18:1_ ω7*c* and/or C_18:1_ ω6*c*), C_19:0_ cyclo ω8*c* and C_18:0_. The DNA G + C content is 63.9 mol%. The type strain is Nit1536^T^ (= KCTC 72347^T^ = NCCB 100697^T^ = JCM 33620^T^), isolated from mangrove sediment of the central Red Sea coast in Thuwal. The GenBank accession number for the 16S rRNA gene sequence of strain Nit1536^T^ is MT146884. The GenBank accession number for the whole genome sequence of strain Nit1536^T^ is CP030941-3.

## Data availability statement

The datasets presented in this study can be found in online repositories. The names of the repository/repositories and accession number(s) can be found in the article/Supplementary material.

## Author contributions

RM, MF, and DD conceived and designed the study and experiments. RM and MF collected the sample. FS, GMe, AB, KS, and RM performed the experiments. RM, GM, CPA, and FS analyzed the data. DD contributed reagents, materials, and analysis tools. RM, GM and DD wrote the manuscript. All authors critically revised the manuscript.

## Footnotes

1
https://github.com/tseemann/abricate

2
https://github.com/kblin/ncbi-genome-download

3
https://lpsn.dsmz.de/search?word=Nitratireductor

4
https://lpsn.dsmz.de

5
https://lpsn.dsmz.de/species/nitratireductor-mangrovi
