# Supplementary material for: The identification of the new species Nitratireductor thuwali sp. nov. reveals the untapped diversity of hydrocarbon-degrading culturable bacteria from the arid mangrove sediments of the Red Sea
Source: Front Microbiol. 2023 May 2;14:1155381. doi: 10.3389/fmicb.2023.1155381 (PMC10185800; doi:10.3389/fmicb.2023.1155381)
Supplement: Supplementary file 1 [file Data_Sheet_1.PDF]

Supplementary Material of

Diversity of hydrocarbon-degrading culturable bacteria from the arid mangrove sediments of the Red Sea and identification of the new species *Nitratireductor thuwali* sp. nov.

Ramona Marasco[1]*, Grégoire Michoud[1], Fatmah O. Sefrji[1], Marco Fusi[1], Chakkiath P. Antony [1], Kholoud A. Seferji[1], Alan Barozzi[1], Giuseppe Merlino[1], Daniele Daffonchio[1]*

[1]Red Sea Research Center (RSRC), Biological and Environmental Sciences and Engineering Division (BESE), King Abdullah University of Science and Technology (KAUST), Thuwal, Saudi Arabia

*Corresponding authors: Daniele Daffonchio, King Abdullah University of Science and Technology (KAUST), Red Sea Research Center (RSRC), Thuwal, Saudi Arabia, phone: +966(2)8082884, e-mail: daniele.daffonchio@kaust.edu.sa; Ramona Marasco, King Abdullah University of Science and Technology (KAUST), Red Sea Research Center (RSRC), Thuwal, Saudi Arabia, phone: +966(2)8082884, e-mail: ramona.marasco@kaust.edu.sa.

Supplementary Methods

Supplementary Method S1. Plant growth promoting assay under salinity stress. Seeds of barley (*Hordeum vulgaris* L.) were sterilized with 3% sodium hypochlorite for 5 min and then washed with distilled water and germinated in plates covered with filtered paper at 25°C for 5 days. To determine the maximum salinity concentration that barley can tolerate, we tested different dilutions of Filtered Sea Water (FSW) to obtain 1%, 2%, and 4% of salinity; tap water was used as a positive control (0% salinity). Seeds germination was strongly inhibited by FSW (4% salinity), thus, diluted FSW (2% salinity) was used for further experiments. Pots experiments were conducted to evaluate the growth-promoting effect of bacterial strains on barley under salt-stress conditions. Germinated seeds were sown in pots containing 205 g of a mixture of sand and organic-rich soil (1:1 v/v). Ten seeds were sown in each pot and the experiment was carried out with five replicates. Pots with the germinated plantlets were treated with 10^9 bacterial cells and pots were irrigated with diluted FSW (2% salinity) every 2 days and kept under conditions of 12 h of photoperiod and a temperature of 25°C in the greenhouse (KAUST, Thuwal, Saudi Arabia). To avoid the accumulation of salt in soil (over 2%), every 5 days, plants were irrigated with tap water. Two groups of untreated seeds were used as control, one irrigated with tap water (positive control) and the other with diluted FSW (negative control). After three weeks of the experiment, plants were harvested and growth parameters were recorded, *i.e.*, fresh weight of shoots and roots. After measurements, plants were incubated in an oven at 50°C until biomass weight was stable and dry weight was recorded for both shoot and root portions. Statistical differences in fresh and dry biomasses between treatments were evaluated using ANOVA test at ($p < 0.05$) and multi-comparison tests.

Supplementary Result

Supplementary Result S1. Metabolism-related features inferred from Nit1536^T genome.

<u>*Carbon and energy pathways*</u>

- All subunits of Cytochrome bc1 complex and NADH:quinone oxidoreductase were encoded. This would enable aerobic respiration in the isolate. Components of F-type ATPase (complex V) were also predicted in the genome.
- The two core subunits (CydA and CydB) of cytochrome bd oxidase but not the associated subunits were encoded on the genome. If the high affinity cytochrome bd oxidase is functional in the isolate, it would not only facilitate the survival of this strain under oxygen-poor conditions (microaerophilic) but would also confer it enhanced tolerance to nitrosative stress, resistance to hydrogen peroxide, suppression of extracellular superoxide production and the ability to defend against antibacterial agents.
- A complete TCA cycle is encoded implying that heterotrophic carbon compounds (for example, organic acids, polycyclic aromatic hydrocarbons, etc.) can be used as sole carbon and energy sources. For complete list of key PAH-degrading genes that were identified in the genome, see the Supplementary Table S3A.
- Anaplerotic CO_2 fixation for replenishment of TCA cycle intermediates could be mediated through the encoded pyruvate carboxylase or phosphoenolpyruvate carboxykinase.
- Almost all metabolic pathways for sugar utilization are encoded on the genome i.e., the glycolysis (EMP), Entner-Duodoroff (EDD) and the Pentose-phosphate pathways.
- Autotrophic CO_2 fixation capacity is likely absent due to the absence of some of the key genes for CBB pathway, reverse Krebs cycle, Wood-Ljunghdhal pathway, Dicarboxylatehydroxybutyrate cycle, Hydroxypropionate-hydroxybutylate cycle and the 3-hydroxypropionate cycle (3-HP).
- Growth on acetate or C2-compounds as a sole source of carbon and energy is possible since the genome encodes acetyl-CoA synthetase (that would feed acetate directly into the TCA cycle), acetate transporter (ActP), as well as enzymes from the Glyoxylate shunt (isocitrate lyase and malate synthase). The presence of the latter pathway along with the key genes encoding enzymes of the beta-oxidation pathway (acyl-coA dhydrogenase, enoyl-CoA hydratase, 3-hydroxyacyl-CoA dehydrogenase, 3-ketoacyl-CoA thiolase) could confer the ability to grow on fatty acids.
- Presence of formate dehydrogenases and components of the serine cycle may imply that the isolate possesses the capacity to detoxify and utilize formate as a source of carbon and/or energy.
- No phototrophic modules are present and no lithotrophic modules for energy generation via ammonia (no amoCAB), hydrogen (no Fe hydrogenases or Fe-Fe hydrogenases or Ni-Fe hydrogenases), and sulphur are present (no fccB, sqr, dsrA,B,D, sdo, sor, sreA,B,C, soxBC, asrA,B,C, aprA, sat or phsA; soxYZ was present though).
- Although an aerobic carbon-monoxide dehydrogenase (CODH) was annotated on RAST and DRAM to permit the use of carbon monoxide (CO) as an additional source of energy, this might constitute a misannotation as the amino-acid sequences of the predicted subunits only showed <55% identity to reference sequences of bonafide CODH (for e.g., WP_013913730.1, WP_003892166.1 and WP_006067999.1).
- Glycerol could serve as a carbon and energy source. By the action of the enzymes glycerol kinase and glycerol-3-P dehydrogenase and glycerol-3-phosphate ABC transporters UgpACE,

dihydroxyacetone phosphate may be formed which could then be acted upon by the enzymes from the bottom half of the glycolysis pathway (all the relevant enzymes are encoded- Triose-P-isomerase, glyceraldehyde-3-P dehydrogenase, phosphoglycerate kinase, phosphoglycerate mutase, enolase, pyruvate kinase and pyruvate dehydrogenase) to ultimately form acetyl-coA that gets fed into the TCA cycle. Glycerol, in this way, could be used as a sole C and energy source.

- No pathways for methanogenesis or methanotrophy were found.
- Apart from alcohol dehydrogenases and a putative lactate dehydrogenase, fermentative pathways for pyruvate (involving for example, pyruvate formate lyase, pyruvate ferredoxin oxidoreductase), acetate (involving for example, acetaldehyde dehydrogenase), butyrate, propionate & hydrogen production (via NAD-reducing hydrogenase) appears to be absent.
- All potential CAZymes as predicted by DRAM tool were listed in Supplementary Table S3B.
- The cellulose degradation pathway was predicted to be only 66% complete on gapseq tool and hence, the isolate appears to lack this ability.
- For chitin degradation, only the chitin deacetylation step was predicted to be complete but not any of the other steps involved. Similarly, gapseq predicted the lignin, pectin, starch and mucin degradation pathways to be incomplete.
- The genome does not show genes encoding the key enzymes for the anaerobic degradation of hydrocarbons- alkylsuccinate synthase, alkane C2-methylene hydroxylase, benzene carboxylase, TutF, ethylbenzene dehydrogenase, phenylethanol dehydrogenase, acetophenone carboxylase, p-cymene dehydrogenase, 2-napthylmethylsuccinate synthase, naphthalene carboxylase, phenylphosphate synthase and carboxylase, hydroxybenzylsuccinate synthase, and p-cresol methylhydroxylase.

Nitrogen metabolism

- Only genes encoding the small and large subunits of a putative assimilatory nitrite reductase (nirBD) and the assimilatory nitrate reductase (nas) were predicted in the genome.
- Genes involved in nitrogen fixation (for e.g., nifH, vnfD), denitrification (nitrous oxide reductase, nitric oxide reductase), dissimilatory nitrite reduction to ammonia (nrfA), nitrate reduction (napA), nitrite reduction (nxrAB), bacterial anaerobic ammonia oxidation (hzsC), and aerobic bacterial ammonia oxidation (amoCAB, hao) were absent.
- Genes involved in urea uptake (ABC transporters, urtBCDE) and assimilation (urease-ureABCDEFG) were encoded.
- Glutamine synthetase (GlnA) and glutamate synthase (gltBD) for assimilating ammonium were encoded on the genome. Glutamate dehydrogenase (GdhA), the alternative enzyme for ammonium assimilation, appeared to be absent in the genome.

Transporters

ABC transporters for peptides/amino-acids, urea, manganese and diverse (non-specific) organic substrates and sugars, cation transporters for magnesium, nickel, cobalt and copper (lead/cadmium/zinc/mercury-translocating P-type ATPase encoded), transporters for ammonium, phosphates (nptA; phosphate transport system regulatory proteins PhoBU), phosphate transport proteins such as PstSCAB, low-affinity inorganic phosphate transporter Pit and alkaline phosphatases were encoded but phytases and acid phosphatases appear to be absent. VitB12 transporter, TonB-dependent transporters, TRAP carboxylate transporters and MFS permeases for diverse substrates, such as organic acids, were predicted by RAST. Sugar phosphotransferase systems (PTS) were not predicted. FeGenie tool did not identify any genes for iron transport, heme

transport, siderophore synthesis or transport. A putative biosynthetic gene cluster for siderophore though was predicted in the genome by the antiSMASH tool via the pHMM detection module.

Motility

Although several genes for flagellar synthesis were observed, the genes encoding for chaperone proteins FlgN, FliS, FliT, the filament cap FliD, regulatory proteins FlhC and FlhD and the biosynthesis proteins FliO and FliH appeared to be absent from the genome.

Stress response, virulence and resistance-related mechanisms

- AMRFinderPlus tool identified putative antibiotic resistance genes that had >80% query coverage and shared 56-64% identity at the amino acid level to class A beta-lactamases (bla) and fosfomycin resistance hydrolase (fosX).
- No CRISPR-Cas systems were identified on RAST or upon running CRISPRCasTyper and DefenseFinder tools. DefenseFinder tool identified the antiphage defense systems Gabija, AbiEII, and Type I Restriction-modification (RM).
- Screening of the genome on ABRicate against the Virulence Factor Database (VFDB) identified only one potential virulence factor pertaining to acpXL that encoded a LPS acyl carrier protein.
- No subsystems related to desiccation or acid stress tolerance were identified on RAST.
- Heat-shock proteins pertaining to the dnaK gene cluster and cold-shock proteins pertaining to the CspA family were identified in the genome.
- Catalases, peroxidase and superoxide dismutases (sodA, sodC) were encoded on the genome. In addition to these oxidative stress mechanisms, as stated earlier, the cytochrome bd oxidase (if functional) would not only facilitate the survival of this strain under microaerophilic conditions but would also confer it enhanced tolerance to nitrosative stress, resistance to hydrogen peroxide, suppression of extracellular superoxide production and the ability to defend against antibacterial agents.
- While copper resistance genes (copA, copB, copF, pcoA, pcoC, and pcoD), heavy metal resistance genes (czcA, czcC, czcD), arsenate resistance (arsB), nickel resistance (nccA), lead resistance (pbrT), gold-copper resistance (golT), cobalt-magnesium resistance (mgtA), and chromate resistance (chrB) genes could not be confirmed, putative arsenate resistance (arsC) and cobalt-nickel resistance (nreB) proteins, a lead, cadmium, zinc and mercury transporting ATPase, along with acriflavine resistance, and cobalt-zinc-cadmium resistance proteins were identified on RAST.

DNA repair

Genes pertaining to DNA repair components such as uvrABC, recAFORQJ, uracil-DNA glycosylases (udg), mutS, mutL, endonucleases (end), and ligases (lig) were found in the genome.

Synthesis of secondary metabolites and pigments

In addition to the identification of an ectoine biosynthesis gene cluster, and putative gene clusters for bacteriocin and siderophore synthesis, antiSMASH tool predicted gene clusters for synthesis of terpene and homoserine lactone. Genes for the biosynthesis of photosynthetic pigments or pigments such as violacein (for example, vioB) were not identified. AntiSMASH also did not identify any biosynthetic gene clusters encoding non-ribosomal peptide synthetases (NRPS) that may be linked with the production of pigments, such as indigoidine.

Synthesis of amino acids

Analysis of predicted proteins on GapMind revealed that the bacterium can synthesize almost all the amino acids (only argD in arginine biosynthesis pathway was predicted with low confidence with the rest of all genes being predicted with high confidence).

Synthesis of osmolytes

- Key genes for the synthesis of glycine beatine were found- choline transporter (betT), choline dehydrogenase (betA), betaine aldehyde dehydrogenase (betB), and cholinesensing repressor (betI).
- Key genes for the synthesis of glycerol were present- glycerol kinase (glpK), glycerol 3-phosphate dehydrogenase (glpD) and a putative phosphoglycerol transferase were identified.
- Synthesis of trehalose- key genes such as trehalose synthase (treS) and trehalosephosphatase (otsB) were present.
- Synthesis of ectoine- key genes encoding L-2,4-diaminobutyric acid acetyltransferase, aspartokinase, diaminobutyrate-pyruvate aminotransferase and ectoine synthase were present.
- Synthesis of glutamate- key genes such as gltB, gltD were found.
- Absent- synthesis of sorbitol (key gene sorbitol dehydrogenase appears to be absent) and synthesis of sucrose (no genes encoding sucrose phosphate synthase and sucrose phosphate phosphatase were found).

Synthesis of storage granules

- Possesses the genetic capacity to synthesize storage granules such as PHA (genes encoding PHA synthase [phaC], 3-ketoacyl-CoA thiolase [phaB], 3-hydroxy-acyl-CoA dehydrogenase (phaA) and PHB depolymerase were found) & polyphosphate (key genes- polyphosphate kinase [ppk] and exopolyphosphatase [ppx] were present) but not glycogen (key gene encoding glycogen synthase glgA was absent).
- FeGenie tool did not identify any genes for iron storage or magnetosome formation.

Supplementary Tables

Supplementary Table S1. List of culturable bacteria harbored by mangrove sediments obtained using the three media, namely MSM, ONR7a and FSW. Bacterial isolates are grouped and classified based on ITS haplotype. For each ITS haplotype, one strain was used to sequence the 16S rRNA and assign taxonomy. Closest describe relative from BLAST identification, percentage of identity whit the related closest relative, and numbers of isolates assigned to each ITS haplotype are also reported.

Medium	Haplotype representative closest described relative	Identity (%)	N. isolates
**MSM**	*Sphingomonas echinoides*	99.72	3
	Sphingobacterium thalpophilum	98.77	1
	Chryseobacterium hagamense	99.45	1
	Pseudomonas parafulva	99.86	10
	Saccharibacillus kuerlensis	97.69	1
	Cupriavidus basilensis	100.00	2
	Pseudomonas plecoglossicida	99.73	3
	Cronobacter sakazakii	99.78	8
	Pseudomonas argentinensis	99.73	1
	Pseudomonas hibiscicola	99.78	2
	Enterobacter cloacae	99.87	1
	Bordetella muralis	99.93	2
	Pseudomonas balearica	99.39	1
	Sphingobium hydrophobicum	97.90	15
	Rhizobium pusense	98.81	1
	Agrobacterium fabrum	99.93	1
	Acinetobacter calcoaceticus	100.00	2
**ONR7a**	*Halomonas halophila*	99.65	2
	Cobetia litoralis	98.94	1
	Joostella atrarenae	97.95	1
	Sinomicrobium oceani	97.75	2
	Pseudoalteromonas arabiensis	97.92	4
	Marinobacter gudaonensis	98.83	2
	Marinobacter salsuginis	97.41	3
	Bacillus megaterium	100.00	2
	Halomonas shengliensis	97.87	1
	Marinobacter adhaerens	97.94	13
	Idiomarina piscisalsi	99.64	1
	Microbulbifer celer	98.46	1
	Halomonas rifensis	97.29	1
	Pelagibaca abyssi	97.84	1
	Halomonas alkaliphila	99.72	2
	Saccharospirillum impatiens	97.05	1
	Pseudoalteromonas donghaensis	99.77	8
	Halomonas aquamarina	97.17	4
	Vibrio alginolyticus	99.09	4
	Marinobacter vinifirmus	97.15	4
	Bacillus simplex	99.87	2
**FSW**	*Halomonas gudaonensis*	98.64	2
	Marinobacter lipolyticus	98.98	2
	Nitratireductor soli	98.94	1
	Halomonas anticariensis	99.72	1
	Thalassospira australica	99.86	7

Marinobacter adhaerens	99.93	2
Chromohalobacter israelensis	100.00	1
Halomonas aquamarina	99.86	6
Idiomarina aquatica	97.46	4
Marinobacter algicola	100.00	2
Pseudoalteromonas donghaensis	100.00	4
Nitratireductor pacificus	**95.41**	**4**
Cobetia pacifica	99.66	2
Halomonas desiderata	98.45	3
Bacillus litoralis	98.46	2
Pseudomonas stutzeri	99.89	5
Micrococcus luteus	98.83	4
Rhodobacter johrii	97.33	1
Salinicola salarius	100.00	1
Roseivivax atlanticus	100.00	2
Marinobacter sediminum	99.59	6
Alcanivorax jadensis	99.57	18
Muricauda aquimarina	98.02	1
Thalassospira profundimaris	99.72	3
Halomonas shengliensis	98.90	1
Reinekea blandensis	98.86	3

Supplementary Table S2. Colony forming unit (CFU) measured for the different media used to isolate the cultivable portion of bacteria from mangrove sediments in presence of crude oil.

**Plate**	**Medium**		
	**ONR7a**	**MSM**	**FSW**
A	1.07×10^5	1.13×10^8	5.11×10^6
B	4.27×10^6	3.98×10^8	7.40×10^6
C	1.25×10^7	3.15×10^7	1.90×10^6

Supplementary Table S3. Genome analysis (**A**) List of key PAH-degrading genes that were identified in the genome of Nit1536^T. (**B**) List of CAZymes as predicted by DRAM tool. See excel file Marasco et al 2022_Supplementary Table S3.

Supplementary Table S4. Metabolic profiling of Nit1536^T strain on Biolog PM9 microplate. Active growth measured as NADH production during cell respiration was reported as positive, ++; weakly positive, +; negative, -.

Well	Substrate	Growth*	Well	Substrate	Growth*
A1	NaCl1%	++	E1	Sodium formate 1%	-
A2	NaCl2%	++	E2	Sodium formate 2%	-
A3	NaCl3%	++	E3	Sodium formate 3%	-
A4	NaCl4%	++	E4	Sodium formate 4%	-
A5	NaCl5%	++	E5	Sodium formate 5%	-
A6	NaCl5.5%	++	E6	Sodium formate 6%	-
A7	NaCl6%	++	E7	Urea 2%	-
A8	NaCl6.5%	++	E8	Urea 3%	-
A9	NaCl7%	++	E9	Urea 4%	-
A10	NaCl8%	++	E10	Urea 5%	-
A11	NaCl9%	+	E11	Urea 6%	-
A12	NaCl10%	+	E12	Urea 7%	-
B1	NaCl6%	++	F1	Sodium Lactate 1%	-
B2	NaCl6%+ Betaine	++	F2	Sodium Lactate 2%	-
B3	NaCl6%+ N-N Dimethyl glycine	++	F3	Sodium Lactate 3%	-
B4	NaCl6%+ Sarcosine	++	F4	Sodium Lactate 4%	-
B5	NaCl6%+ Dimethyl sulphonyl propionate	++	F5	Sodium Lactate 5%	-
B6	NaCl6%+ MOPS	++	F6	Sodium Lactate 6%	-
B7	NaCl6%+ Ectoine	++	F7	Sodium Lactate 7%	-
B8	NaCl6%+ Choline	++	F8	Sodium Lactate 8%	-
B9	NaCl6%+ Phosphoryl choline	++	F9	Sodium Lactate 9%	-
B10	NaCl6%+ Creatine	++	F10	Sodium Lactate 10%	-
B11	NaCl6%+ Creatinine	++	F11	Sodium Lactate 11%	-
B12	NaCl6%+ L-Carnitine	+	F12	Sodium Lactate 12%	-
C1	NaCl6%+KCl	++	G1	Sodium Phosphate pH 7 20 mM	++
C2	NaCl 6 % + L-proline	++	G2	Sodium Phosphate pH 7 50 mM	++
C3	NaCl 6 % + N-Acethyl L-glutamine	++	G3	Sodium Phosphate pH 7 100 mM	++
C4	NaCl 6 % + ß-Glutamic acid	++	G4	Sodium Phosphate pH 7 200 mM	++
C5	NaCl 6 % + γ-Amino-n-butyric acid	++	G5	Sodium Benzoate pH 5.2 20 mM	-
C6	NaCl 6 % + Glutathione	++	G6	Sodium Benzoate pH 5.2 50 mM	-
C7	NaCl 6 % + Glycerol	++	G7	Sodium Benzoate pH 5.2 100 mM	-
C8	NaCl 6 % + Trehalose	++	G8	Sodium Benzoate pH 5.2 200 mM	-
C9	NaCl 6 % + Trimethylamine-N-oxide	++	G9	Ammonium sulfate pH 8 10 mM	-
C10	NaCl 6 % + Trimethylamine	++	G10	Ammonium sulfate pH 8 20 mM	-
C11	NaCl 6 % + Octopine	++	G11	Ammonium sulfate pH 8 50 mM	-
C12	NaCl 6 % + Trigonelline	+	G12	Ammonium sulfate pH 8 100 mM	-
D1	Potassium chloride 3%	++	H1	Sodium Nitrate 10 mM	++
D2	Potassium chloride 4%	+	H2	Sodium Nitrate 20 mM	++
D3	Potassium chloride 5%	-	H3	Sodium Nitrate 40 mM	++
D4	Potassium chloride 6%	-	H4	Sodium Nitrate 60 mM	++
D5	Sodium sulfate 2%	++	H5	Sodium Nitrate 80 mM	++
D6	Sodium sulfate 3%	++	H6	Sodium Nitrate 100 mM	++
D7	Sodium sulfate 4%	++	H7	Sodium Nitrite 10 mM	+
D8	Sodium sulfate 5%	++	H8	Sodium Nitrite 20 mM	-
D9	Ethylene glycol 5%	++	H9	Sodium Nitrite 40 mM	-
D10	Ethylene glycol 10%	++	H10	Sodium Nitrite 60 mM	-
D11	Ethylene glycol 15%	++	H11	Sodium Nitrite 80 mM	-
D12	Ethylene glycol 20%	+	H12	Sodium Nitrite 100 mM	-

*Measured as reduction a tetrazolium dye consequently to cell respiration (*i.e.*, NADH production)

Supplementary Table S5. Antibiotic and inhibitor compounds sensitivity test of Nit1536^T on Biolog PM11 and PM12 microplates. Strain sensitivity to antibiotics/inhibitors is scored; Biolog PM plates contain four different concentrations for each compound from lower [well 1/4] to highest [well 4/4]; information regarding the concentrations of compounds in each well is not available from the company. We classified the sensitivity/resistance capacity of our strain based on its ability to grow at different concentrations. For instance, antibiotic sensitive (S), *i.e.*, the bacterial growth is inhibited at all the four antibiotic concentrations available in the PM plates; antibiotic resistance (R), *i.e.*, the bacterial grow at all the concentration of the antibiotic; intermediate resistance (IR), *i.e.*, bacteria can grow only in the presence of low concentrations of the antibiotic (1 and 2 wells of the compounds).

**Antibiotic class (Biolog, PM11)**		**Sensitivity**	**Antibiotic class (Biolog, PM12)**		**Sensitivity**
**Aminoglycosides**	Amikacin	R	**Glycopeptides**	Bleomycin	R
	Neomycin	R		Colistin	R
	Gentamicin	R		Capreomycin	R
	Kanamycin	R		Polymyxin B	R
	Paromomycin	R		Vancomycin	R
	Sisomicin	R	**Tetracyclines**	Chlortetracycline	IR
	Novobiocin	S		Minocycline	R
	Tobramycin	R		Demeclocycline	IR
	Spectinomycin	S		Tetracycline	R
**β- lactams**	Amoxicillin	R		Penimepicycline	IR
	Cloxacillin	S	**Amphenicols**	Chloramphenicol	IR
	Nafcillin	S	**Macrolides**	Erythromycin	IR
	Cefazolin	R		Spiramycin	S
	Ceftriaxone	IR	**Sulfonamides**	Sulfamethoxazole	R
	Cephalothin	IR		Sulfathiazole	R
	Penicillin G	R		Sulfadiazine	R
	Carbenicillin	R		Sulfamethazine	R
	Oxacillin	IR	**Rifamycins**	Rifampicin	IR
**Lincosamides**	Lincomycin	IR			
**Synthetic antibiotics**	Lomefloxacin	R	**Others compounds**	Potassium tellurite	R
	Enoxacin	R		Benzethonium chloride	S
	Nalidixic acid	R		D,L-Serine hydroxamate	IR
	Ofloxacin	R		5-Fluoroorotic acid	R
				L-Aspartic-β- hydroxamate	R
				2,4-Diamino-6,7-diisopropylpteridine	IR
				Dodecyltrimethyl ammonium bromide	S

Supplementary Table S6. The Nit1536^T strain was tested as biopromoter and bioprotector for plants, *i.e.*, barley under salt stress. Positive control indicates plants irrigated with tap water; negative control those plants irrigated with diluted FSW (2% salinity) and non-treated with bacteria, and Nit1536^T the plants irrigated with diluted FSW (2% salinity) and treated with a Nit1536^T bacterial cells. Star (*) indicates the parameter positively affected by the treatment with Nit1536^T compared to the negative control.

**Parameter**	**Positive control**	**Negative control**	**Nit1536^T**
Root FW*	0.36 ± 0.08 (c)	0.12 ± 0.05 (a)	0.21 ± 0.06 (b)
Root DW*	0.062 ± 0.018 (c)	0.029 ± 0.013 (a)	0.045 ± 0.015 (b)
Shoot FW	0.62 ± 0.14 (a)	0.49 ± 0.11 (b)	0.51 ± 0.09 (b)
Shoot DW	0.15 ± 0.05 (a)	0.9 ± 0.06 (a)	0.11 ± 0.05 (a)

Supplementary Figures

Supplementary Figure S1. (**A**) Sampling site and (**B**) detail of *Avicennia marina* sediments used as starting inoculum during the process of isolation.

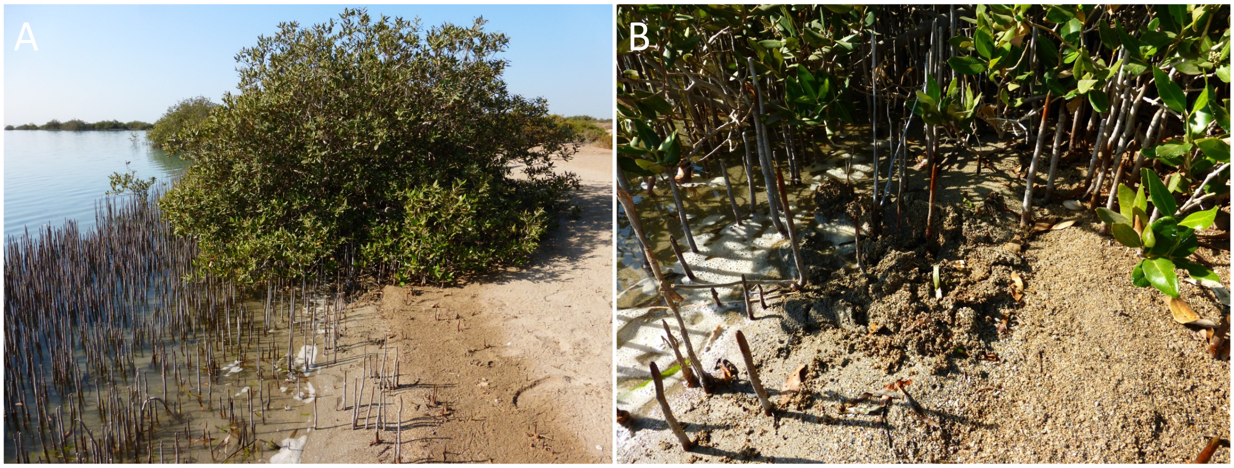

Supplementary Figure S2. Sequence similarity of the 16S rRNA gene from the four strains (Nit1536, Nit1531, Nit1537 and Nit1539) belongs to the ITS haplotype with 95.41% of similarity with *Nitratireductor pacificus* (Supplementary Table S1).

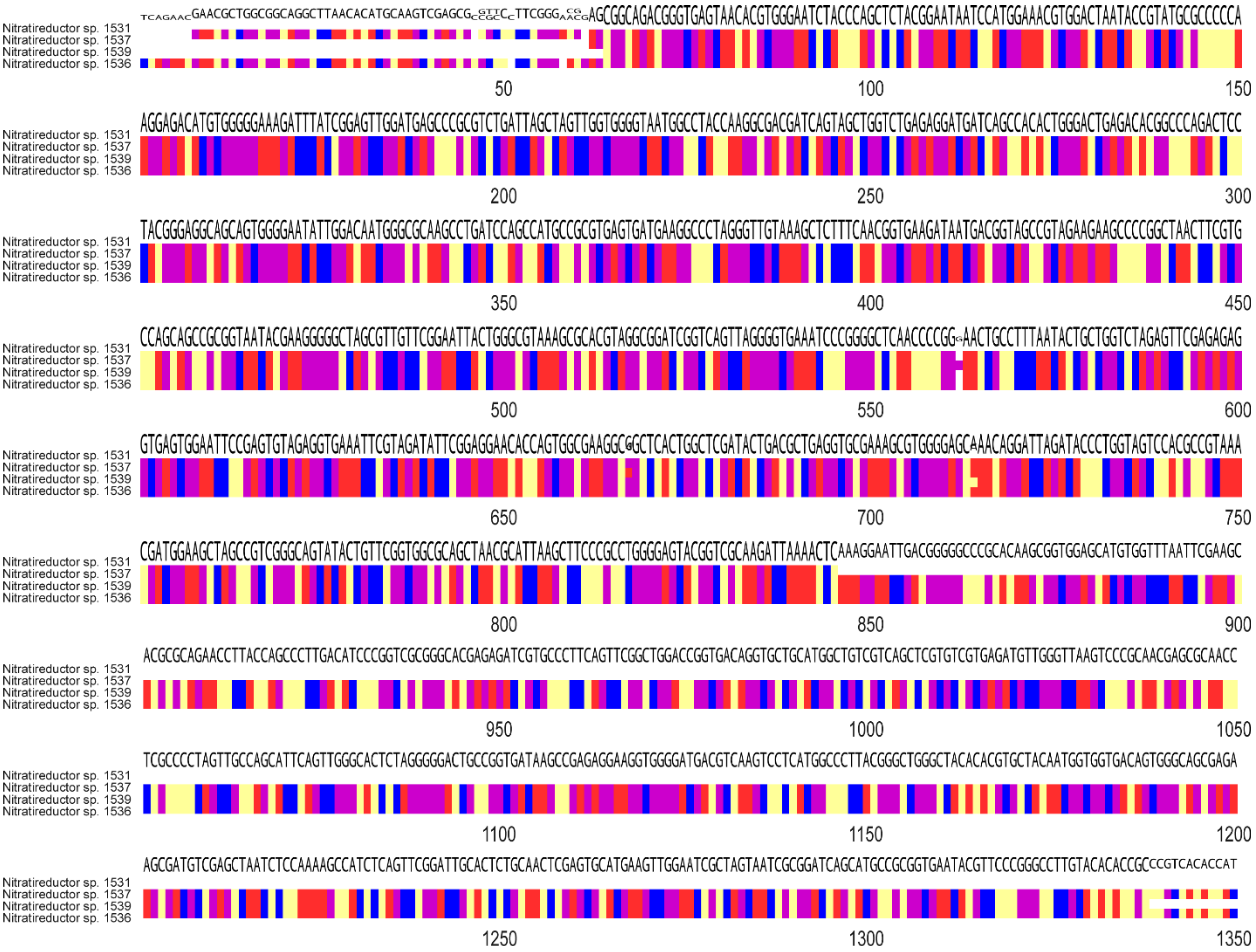

Supplementary Figure S3. Results of Enterobacterial repetitive intergenic consensus (ERIC)-PCR (De Bruijn, 1992) performed on the four isolates within the same ITS haplotype that showed 95.41% of similarity with *Nitratireductor pacificus* (Supplementary Table S1).

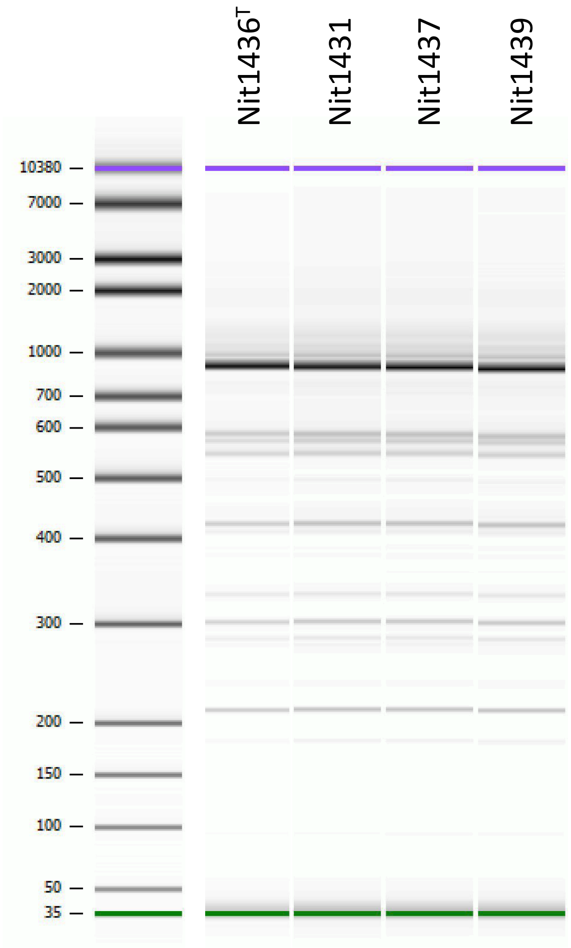

Supplementary Figure S4. Heat map showing the presence and relative abundance of genera across the three different media. The reported percentages are calculated on the total number of isolates for each medium: 55, 60 and 88 from MSM, ONR7a and FSW plates, respectively.

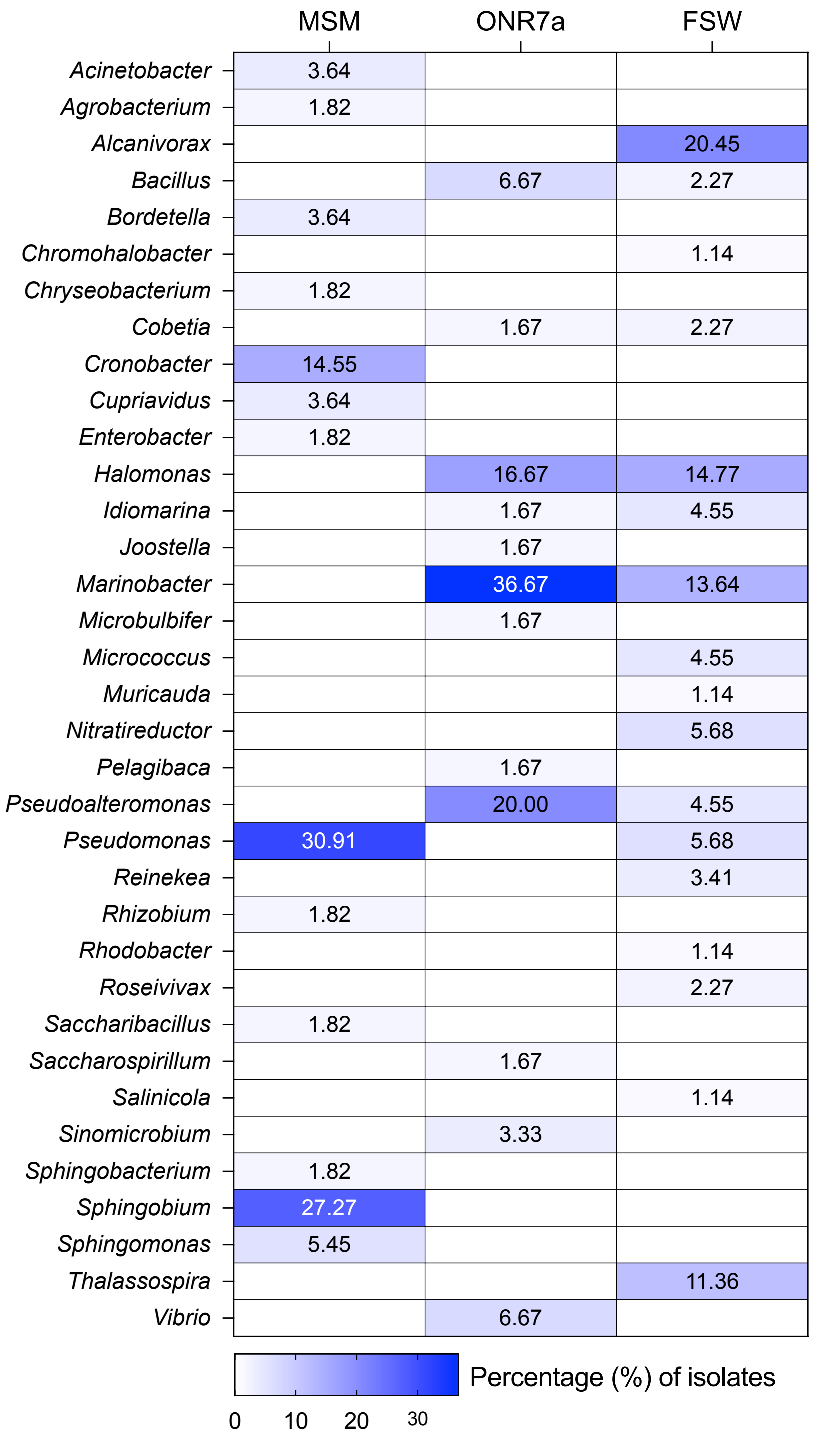

Supplementary Figure S5. Metabolic profiling of Nit1536^{T} strain on Biolog PM1 and PM2 microplates with 190 different carbon sources. For each plate, the active growth measured as NADH production during cell respiration was reported as fold change values respect to the negative control (water) in well A1. Content of each well is also reported

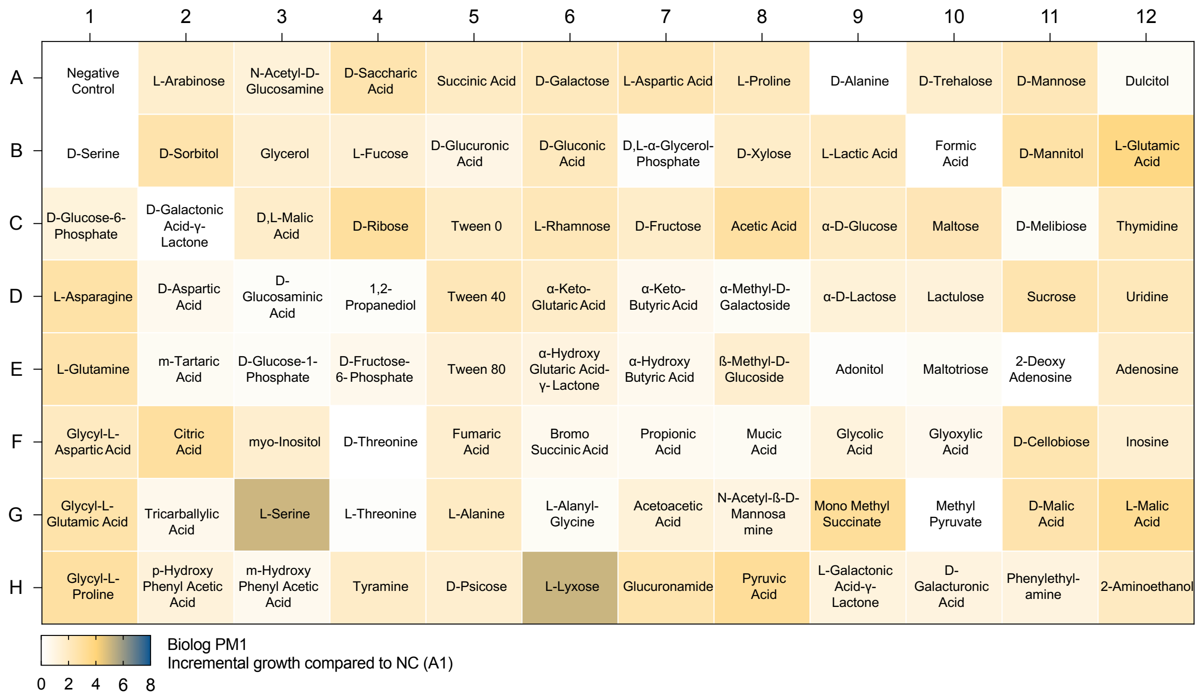

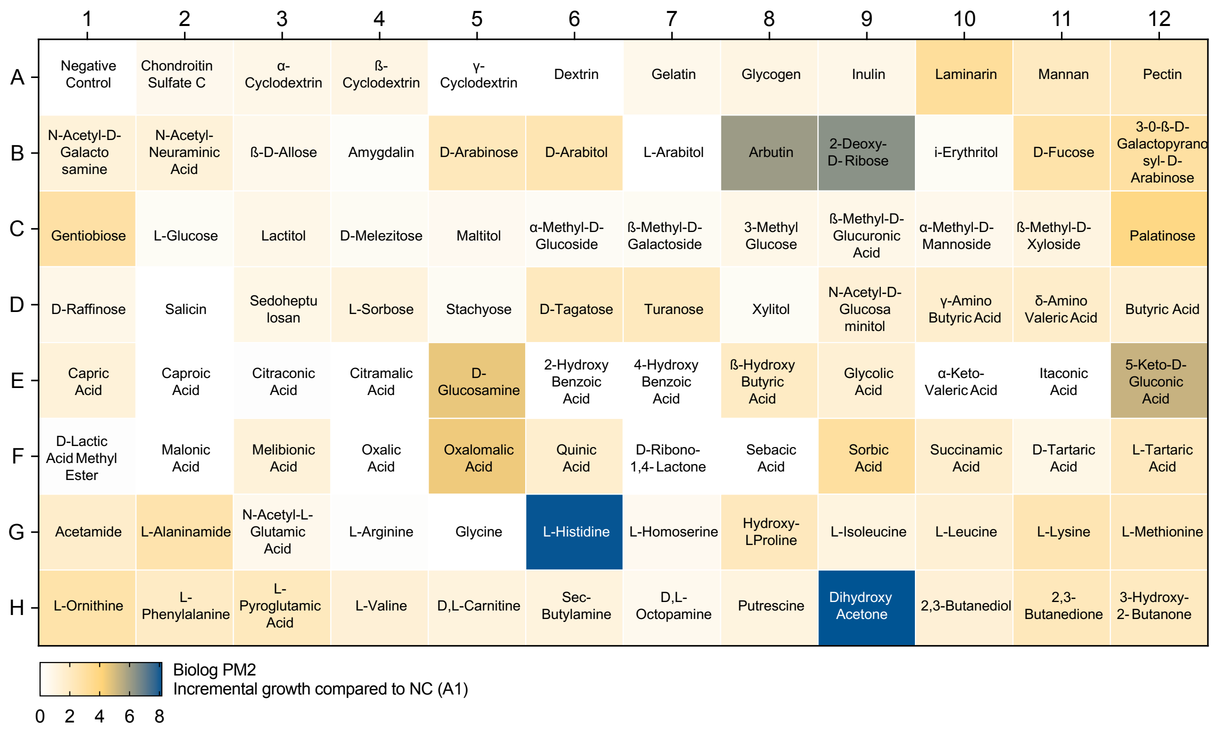

Supplementary Figure S6. Results of the polar lipid identification. For all the strains the analysis was conducted by DSMZ.

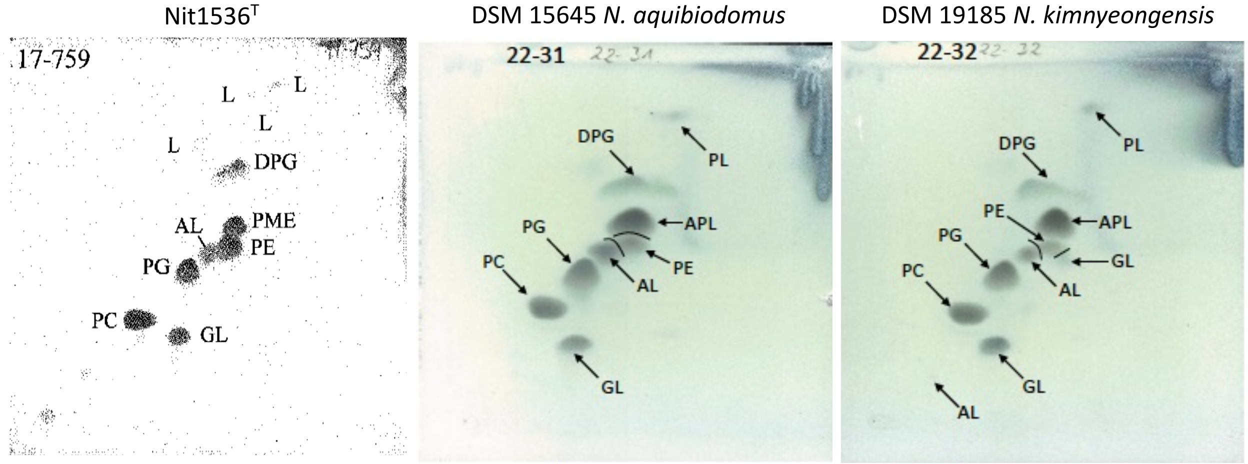

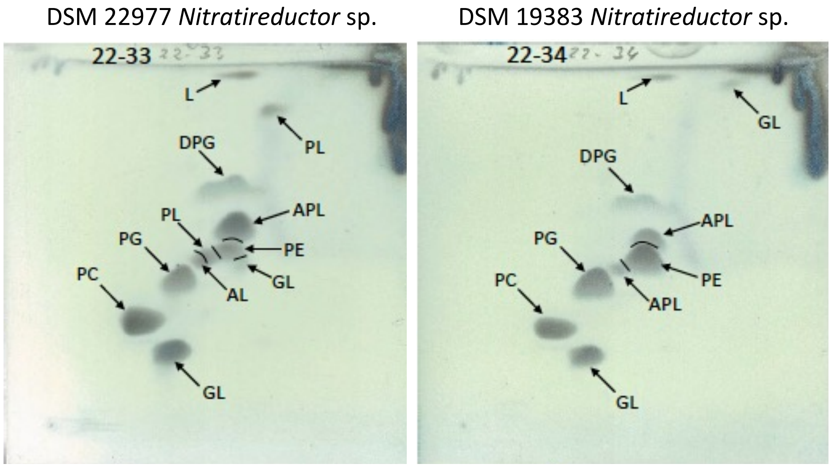

DPG = Diphosphatidylglycerol

PG = Phosphatidylglycerol

PE = Phosphatidylethanolamine

PC = Phosphatidylcholine

APL = Aminophospholipid

AL = Aminolipid

GL = Glycolipid

PL = Phospholipid

L = Lipid

Supplementary Figure S7. Graphical circular map of the chromosome and genome features of *N. thuwali* strain Nit1536^{T}. From outside to the centre: Genes on the forward strand, Genes on the reverse strand, RNA genes (tRNAs purple, rRNAs blue), GC content, GC skew.

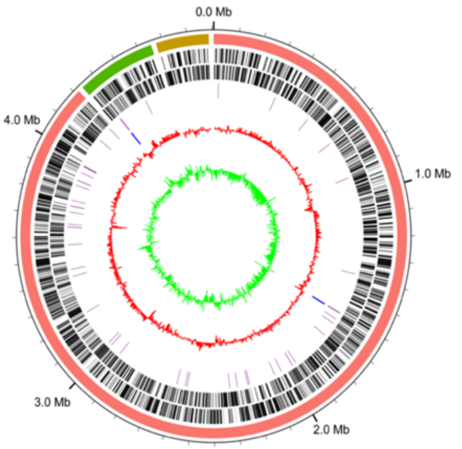

Attribute	value
Genome size (bp)	4,746,780
DNA G+C content (mol%)	63.9
Number of contigs	3
Total genes number	4636
Protein coding genes (CDSs)	4558
rRNA	6
tRNA	54
Other RNA genes	18
Genes with signal peptide	434

Supplementary Figure S8. Extracted HPLC_MS chromatogram of identified osmolytes (m/z 143, 118, 148, 365, 90, 258, 135) classified from the most abundant analyte to the lowest in solution; osmolytes identification: ectoine, betaine, glutamate, proline, trehalose, alanine, glycerophosphorylcholine and dymethylsulfoniopropionate.

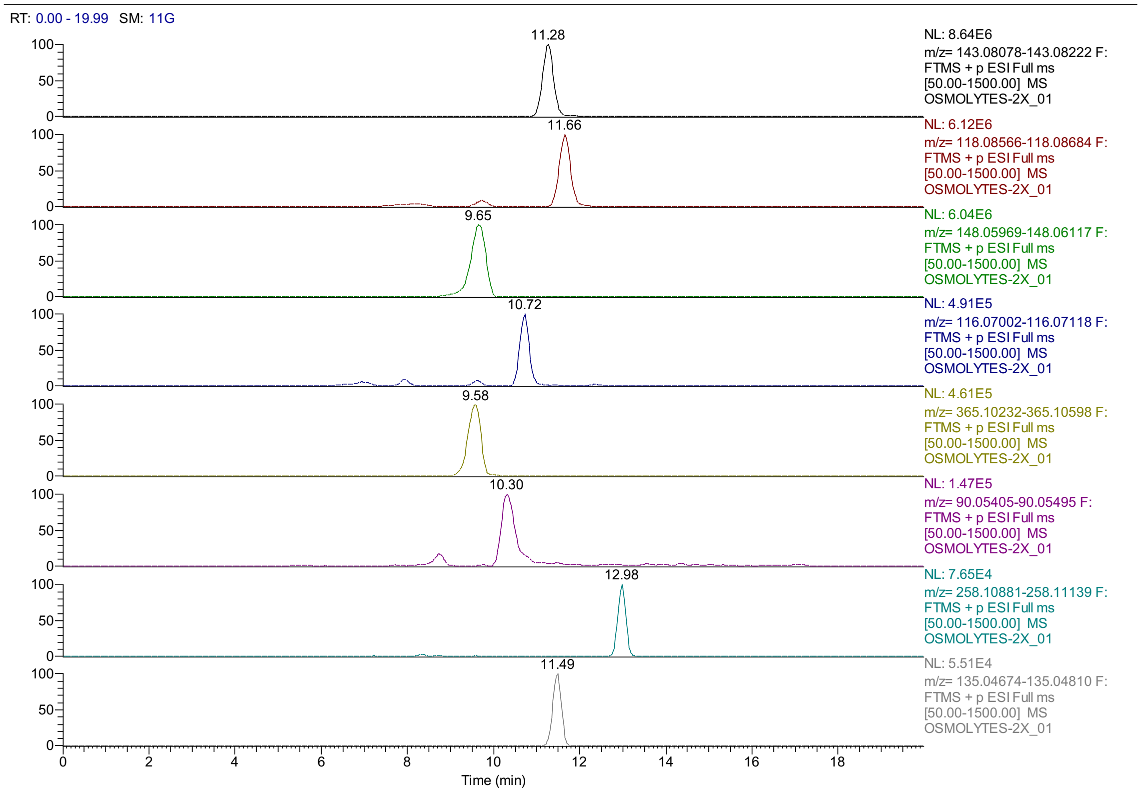

Supplementary References

De Bruijn, F. J. (1992). Use of repetitive (repetitive extragenic palindromic and enterobacterial repetitive intergeneric consensus) sequences and the polymerase chain reaction to fingerprint the genomes of *Rhizobium meliloti* isolates and other soil bacteria. *Appl. Environ. Microbiol.* 58, 2180–2187. doi:10.1128/aem.58.7.2180-2187.1992.
